# Metabolome and Transcriptome Profiling of Chicory Roots Provide Insights Into Laticifer Development and Specialized Metabolism

**DOI:** 10.1111/ppl.70778

**Published:** 2026-02-08

**Authors:** Khabat Vahabi, Gerd U. Balcke, Johanna C. Hakkert, Ingrid M. van der Meer, Benedikt Athmer, Alain Tissier

**Affiliations:** ^1^ Leibniz Institute of Plant Biochemistry Halle (Saale) Germany; ^2^ Wageningen Plant Research Bioscience, Wageningen University & Research Wageningen the Netherlands

**Keywords:** biosynthesis, chicory roots, inulin, latex, laticifer, sesquiterpene lactone, transport

## Abstract

Chicory roots produce inulin, a dietary fiber, as well as large quantities of bitter sesquiterpene lactones (STLs), which have valuable biological activities. In an effort to understand the compartmentalization of metabolism within chicory roots and the molecular basis of the development of laticifers that produce the chicory latex, we performed metabolomics and transcriptomics profiling of different tissues of chicory roots. Gas chromatography coupled to mass spectrometry (GC–MS) and liquid chromatography coupled to mass spectrometry (LC–MS) identified a total of 21,437 features, of which 135 were differentially abundant between cell types. Further analysis indicated that the major STLs accumulated primarily in the latex. Gene expression of known STL pathway genes indicates a compartmentalization of the biosynthesis across multiple tissues, with implications regarding the trafficking of pathway intermediates. Phytohormone measurements and gene expression analysis point to a major role for jasmonate signaling in the development and differentiation of laticifers. Furthermore, inulin accumulates mostly outside the laticifers, but expression of inulin metabolic genes also points to a complex distribution and trafficking of inulin or inulin precursors across different root compartments. Altogether, the data presented here constitute a unique resource to investigate several biological processes in chicory roots, including laticifer development, STL biosynthesis and transport, and inulin biosynthesis regulation.

## 1 Introduction

Root chicory ( *Cichorium intybus var. sativum* L.) is a variety of common chicory that is grown for its inulin‐containing roots. Inulin is a soluble fructan polymer with a terminating glucose residue that is used as a dietary fiber and sugar replacement in many food products, such as yogurts, other dairy products and bakery goods. According to the Food and Agriculture Organization (FAO), gross production value of root chicory is about 500 million US dollars per year (Table 1). Most of the production of root chicory comes from Europe, with Belgium, France, the Netherlands and Poland ranked as the main producers (Table 1).

**TABLE 1:** Worldwide production and value of chicory root production (in 2017^a^). (*Source:* FAOSTAT https://www.fao.org/faostat/en/#home).

Country	Production (in t)	Value (1000 $ US)
Belgium	327760.00	230,828
Netherlands	54800.00	38,593
France	50335.40	35,449
Poland	29130.00	20,515
South Africa	6566.00	4624
Ukraine	5803.33	4087
Rest of the World	30980.75	16,505
Total	505626.53	350,601

*Note:* ^a^2017 was the last year with reliable data for chicory root production available at FAOSTAT.

**FIGURE 1:**
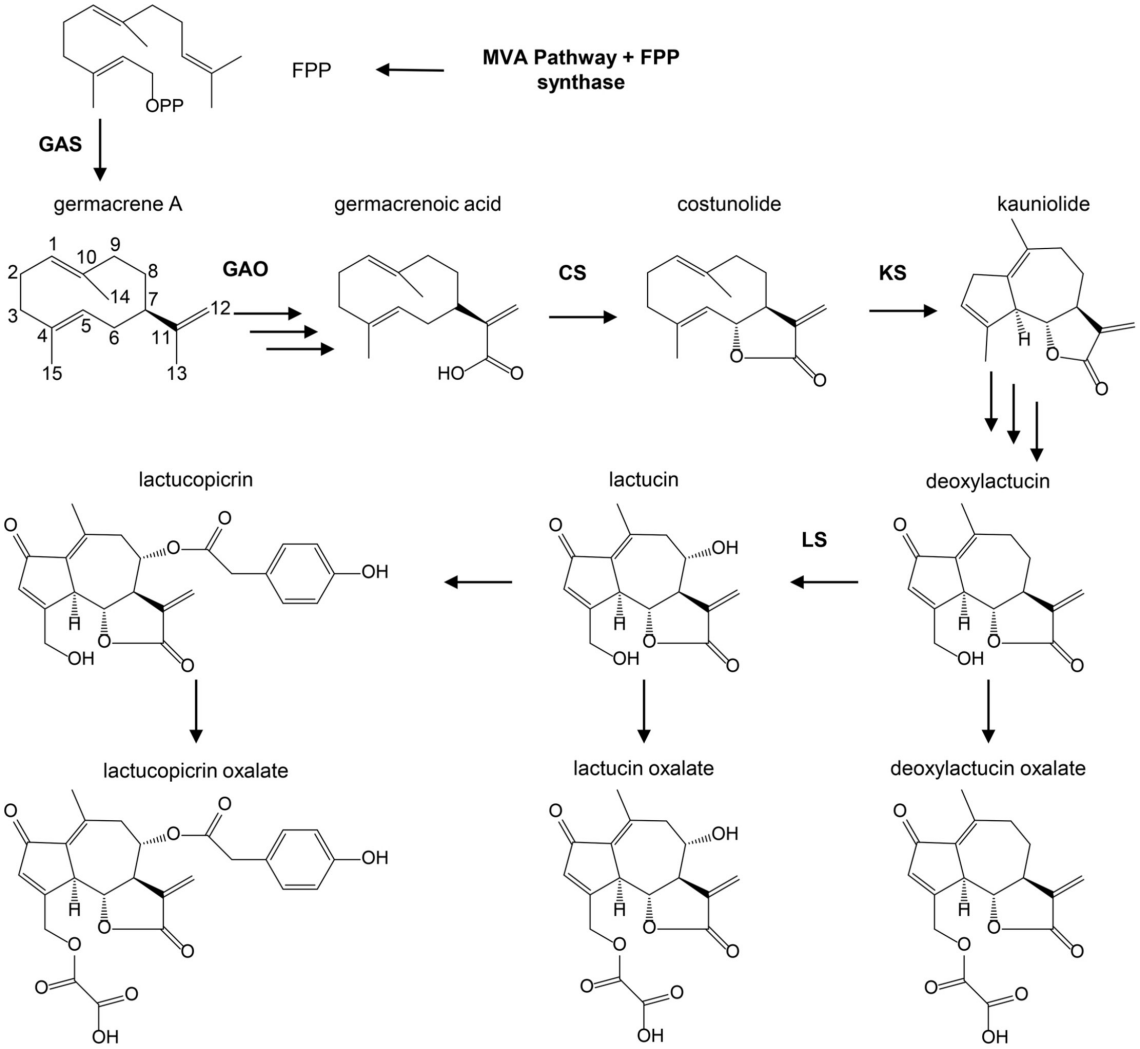
Biosynthesis pathway of sesquiterpene lactones in chicory. The known enzymatic steps are abbreviated as follows: COS: Costunolide synthase; GAS: Germacrene A synthase; GAO: Germacrene A oxidase; KS: Kauniolide synthase; LS: Lactucin synthase; FPP: Farnesyl diphosphate.

A traditional use of root chicory that goes back to the 18th and 19th centuries in Germany and France is as coffee replacement. Although chicory does not contain any caffeine, it has bitter compounds that confer a similar taste to coffee. These bitter compounds are sesquiterpene lactones (STLs), which occur frequently in the Asteraceae family. In many plants of the Asteraceae family, the STLs accumulate to high levels in glandular trichomes or in latex, which is a secretion of the laticifer cells. Chicory roots produce an abundant latex containing the STLs that are derived from germacrene A and include 8‐deoxylactucin (DL), lactucin, lactucopicrin and their oxalate conjugates (de Kraker et al. 1998; Figure 1). STLs constitute a large and diverse group of sesquiterpenoids, with various pharmacological and biological activities, including antibacterial, anti‐inflammatory and anti‐helminthic (Li et al. 2018; Woolsey et al. 2019). Chicory STLs also possess anti‐helminthic and anti‐inflammatory activities (Häkkinen et al. 2021; Matos et al. 2020; Peña‐Espinoza et al. 2015; Peña‐Espinoza et al. 2020). Probably the most famous of STLs is artemisinin, a compound with anti‐malarial activity produced in the glandular trichomes of sweet wormwood ( *Artemisia annua* ; Xiao et al. 2016). In only a few cases is the function of STLs in their natural environment known. One example is in dandelion ( *Taraxacum officinale* agg.), where a STL produced in latex, a glycosylated taraxinic acid, confers resistance to insect larvae that feed on the roots (Huber, Bont, et al. 2016; Huber, Epping, et al. 2016).

STLs interfere with the extraction of inulin from chicory roots. Therefore, there is interest in generating chicory lines with reduced STL content. A recent report describes the generation of clustered regularly interspaced short palindromic repeats associated protein 9 (CRISPR/Cas9) knock‐outs of chicory genes encoding germacrene A synthase (GAS; Cankar et al. 2021). All four copies of the chicory *GAS* genes were disrupted, leading to a complete loss of STL production. Interestingly, this was accompanied by increased accumulation of squalene, which derives from the same precursor as the STLs, namely farnesyl diphosphate. On the other hand, because of their potential health benefits and various biological activities, STLs from chicory may also constitute a breeding target, for example to modify the STL content for the accumulation of specific compounds or to globally increase STL productivity in roots. In related *Lactuca* species and in chicory, it was shown that STLs are present in the latex (Sessa et al. 2000). In different plant species, the latex contains high concentrations of various secondary metabolites and plays a protective role against microbes, pests, and wounding by accumulating high levels of toxic proteins and metabolites (Kitajima et al. 2018; Makita et al. 2017). Thus, knowing which genes are involved in the biosynthesis of STLs or in the development of laticifers, and using CRISPR/Cas9 gene editing, it should be possible to rapidly breed novel varieties that have an altered STL profile or with a reduced or increased laticifer volume. For example, inactivation of the genes encoding kauniolide synthase (KLS) resulted in lines accumulating free or conjugated costunolide, a potential anticancer STL (Cankar et al. 2022). For chicory breeding efforts targeting laticifers or the production of metabolites in the laticifers, it is therefore essential to acquire basic information about the metabolite and transcriptome patterns of different plant tissues across developmental stages. However, chicory is not a model species and there are few transcriptomic, metabolomic, and genomic data available until now (Peña‐Espinoza et al. 2020; Testone et al. 2017; Testone et al. 2019). Therefore, in this study we set out to determine the metabolite and transcription profiles of several tissues of chicory roots, including latex, which is the cytosolic compartment of the laticifers. The combined analysis of metabolome and transcriptome data has been very useful to determine key genes and regulatory processes in various plants (Balcke et al. 2017; Cao, Wang, et al. 2017; Verhoeven et al. 2017).

Analysis of the data reported here provides unique insights into the metabolic and developmental processes in chicory taproots. In particular, we could show that STLs are essentially stored in the latex but that their synthesis is compartmentalized across the root tissues, and we identified candidate genes for the biosynthesis and transport of the chicory STLs as well as for the development of laticifers. This study therefore constitutes a foundation for further functional studies of these processes in chicory.

## 2 Materials and Methods

### 2.1 Plant Materials

We used a clone from *Cichorium intybus* (AA, 2*n* = 18, root chicory), Orchies 37 (O37; Cankar et al. 2021) in all the experiments described here and propagated it via tissue culture. For the analysis of the STLs in plant tissues, we used 16‐week‐old plants grown at 25°C, 16‐h photoperiod, and with a light intensity of 80 mol/s/m2 (Figure S1).

### 2.2 Microscopy of the Laticifers and Dissection of the Cell Types

We found that staining with toluidine blue O (TBO) differentiates various cell types. Hand sections of chicory roots (3 mm thickness) were stained with TBO (0.2% in water) for 5 min and washed with distilled water. These stained sections were used as a guide to facilitate the dissection of different cell types in non‐stained sections. Unstained subsequent sections were used for dissecting the hypodermis (HD) and vascular cylinder (VC). The latex (LX) was collected from roots.

### 2.3 Targeted Profiling of the STLs

Different tissue types (HD and VC) and LX from three different biological repetitions were immediately frozen in liquid nitrogen and the time of freezing was the same for all samples. The tissue material (VC and HD) was ground in a mortar and all samples including latex were stored at −80°C. For sample preparation, 100 mg of each sample was transferred to a cryo‐tube (for details see Balcke et al. 2017), and the material was homogenized in a FastPrep instrument using an extraction buffer (80% MeOH, 19.9% H_2_O, 0.1% formic acid) and kept on ice. STLs identification by multiple reaction monitoring (MRM) was carried out by LC–MS/MS analysis of 5 μL of methanolic extracts using an Acquity UPLC (Waters) and a Sciex 6500 QTRAP and the software Analyst 1.7 (Sciex). Peak area quantification of six different STLs was done by the software Multiquant 3.0 (Sciex). The data and MRM parameters are provided in Table S1.

### 2.4 Untargeted Metabolome Profiling

For untargeted metabolite analysis, a modified 2‐phase extraction was performed where 900 μL dichloromethane/ethanol (2:1, −80°C) were first added followed by 150 μL diluted HCl (pH 1.7) to 100 mg of different tissues and extracted using the FastPrep instrument. Phase separation was done by centrifugation (2 min, 20,000 g); the upper phase, containing the hydrophilic metabolites, was collected and stored on ice. Then another 100 μL aqueous HCl was added, and the extraction and phase separation were repeated. Both aqueous extracts were combined and stored at −80°C until the following untargeted analysis of the hydrophilic compounds.

The remaining organic phase was collected and stored on ice, and the tissue debris was re‐extracted with 500 μL tetrahydrofuran (THF). Both organic extracts were combined, dried in a nitrogen stream and dissolved in 80% methanol, centrifuged (2 min, 20,000 g) and analyzed as described in section 2.4.2.

#### 2.4.1 Separation of Hydrophilic Metabolites (hpm)

This was performed on a Nucleoshell RP18 (2.1 × 150 mm, particle size 2.1 μm, Macherey & Nagel GmbH) using a Waters ACQUITY UPLC System, equipped with an ACQUITY Binary Solvent Manager and ACQUITY Sample Manager (10 μL sample loop, partial loop injection mode, 5 μL injection volume, Waters GmbH). Eluents were A (aqueous 10 mmol l^−1^ tributyl amine, adjusted to pH 6.2 with glacial acetic acid) and B (acetonitrile), respectively. Elution was performed isocratically for 2 min at 2% eluent B, from 2 to 18 min with a linear gradient to 36% eluent B, from 18 to 21 min to 95% eluent B and isocratically from 21 to 22.5 min at 95% eluent B, and from 22.51 to 26 min at 2% eluent B. The flow rate was set to 400 μL min^−1^ and the column temperature was maintained at 40°C. Metabolites were detected by negative electrospray ionization and mass spectrometry (MS). The MS detection was done on a QToF‐MS/MS instrument in the negative mode (LC–MS‐hpm; see below).

#### 2.4.2 Separation of Medium Polar Metabolites (mpm)

This was performed on a Nucleoshell RP18 (2.1 × 150 mm, particle size 2.1 μm, Macherey and Nagel GmbH) using a Waters ACQUITY UPLC System, equipped with an ACQUITY Binary Solvent Manager and ACQUITY Sample Manager (20 μL sample loop, partial loop injection mode, 5 μL injection volume, Waters GmbH). Eluents A and B were aqueous solutions of 0.3 mmol l^−1^ NH_4_HCO_2_ (adjusted to pH 3.5 with formic acid) and acetonitrile, respectively. Elution was performed isocratically for 2 min at 5% eluent B, from 2 to 19 min with a linear gradient to 95% B, from 19–21 min isocratically at 95% B, and from 21.01 to 24 min at 5% B. The flow rate was adjusted to 400 μL min^−1^ and the column temperature was maintained at 40°C. Metabolites were detected by positive and negative electrospray ionization using an Acquity UPLC (Waters) and TripleTOF 5600 mass spectrometer. The MS detection of mpm was done on a QToF‐MS/MS instrument in the negative mode and positive modes (LC–MS‐neg and LC–MS‐pos; see below).

In this study, LC–MS‐neg and LC–MS‐pos are abbreviations that stand for measurement of mpm metabolites using QToF‐MS/MS in negative and positive mode, respectively.

#### 2.4.3 Mass Spectrometric Analysis of Small Molecules

This was performed by two strategies: targeted MS/MS via multiple reaction monitoring (QTRAP6500) and untargeted via MS‐TOF‐SWATH‐MS/MS (TripleToF 5600, both AB Sciex GmbH) operating in negative or positive ion mode and controlled by Analyst 1.7.1 and 1.6 TF software (AB Sciex GmbH). The source operation parameters were as follows: ion spray voltage, −4500 V/+5500 V; nebulizing gas, 60 psi; source temperature, 450°C (QTRAP) or 600°C (TripleToF); drying gas, 70 psi; curtain gas, 35 psi. For APCI, a nebulizer current of three units was used. TripleToF instrument tuning and internal mass calibration were performed every five samples with the calibrant delivery system applying APCI negative or positive tuning solutions, respectively (AB Sciex GmbH).

Triple‐ToF data acquisition was performed in MS^1^‐ToF mode and MS^2^‐SWATH mode. For MS^1^ measurements, ToF masses were scanned between 65 and 1250 Da with an accumulation time of 50 ms and a collision energy of 10 V (−10 V). The MS^2^‐SWATH‐experiments were divided into 26 Da segments of 20 ms accumulation time. Together, the SWATH experiments covered the entire mass range from 65 to 1250 Da in 48 separate scan experiments, which allowed a cycle time of 1.1 s. Throughout all MS/MS scans, a declustering potential of 35 (or− 35 V) was applied. Collision energies for all SWATH‐MS/MS were set to 35 V (−35) and a collision energy spread of ±25 V, maximum sensitivity scanning, and otherwise default settings.

### 2.5 Metabolome Annotations

MS‐Dial (v. 4.9) was used to extract and align MS1 and MS/MS features from SWATH‐QToF runs (Tsugawa et al. 2015). For best match spectra annotation of individual mz;r.t.‐features, an in‐house MS/MS database was used in the MS‐Dial environment. Xcalibur software was used to annotate GC–MS data using the NIST17 EI‐MS library. The MetFamily (Treutler et al. 2016) software program was used to annotate groups of features having similar MS/MS spectra in the LC‐QToF‐MS/MS data sets. With unique structural identifiers, such as SMILES supplied by database matching, we generated ChemOnt terms of metabolite families based on the ClassyFire annotation (Djoumbou Feunang et al. 2016). Metabolite functional classification was carried out using the KEGG Mapper (Kanehisa and Sato 2019) online tool, and enrichment Fold changes based on KEGG pathways were carried out according to the ratio of the proportions of the treatment to the background (Huang et al. 2008).

### 2.6 GC–MS Analysis

We mixed 100 mg of LX, HD and VC tissues with 2 mL hexane and after 5 min of centrifugation at 20000 g, the hexane extracts were sealed in the GC vials. 1 μL of extracts was injected directly into the GC–MS for analysis on a Trace GC Ultra gas chromatograph coupled to an ATAS Optic 3 injector and an ISQ single quadrupole mass spectrometer (Thermo Fisher Scientific) with electron impact ionization. The injection temperature rose from 60°C to 250°C at 10°C/s and the flow rate of helium was 1 mL min^−1^. The GC oven temperature ramp was as follows: 50°C for 1 min, 50°C to 300°C with 7°C/min, 300°C to 330°C with 20°C/min, and 330°C for 5 min. Mass spectrometry was performed at 70 eV in full scan mode with *m/z* ranging from 50 to 450. Data analysis and chromatograms were implemented with the device‐specific software Xcalibur (Thermo Scientific).

### 2.7 Plant Hormones Analysis

We analyzed plant hormones according to Balcke et al. (2012). First, we took 50 mg of LX, HD, and VC tissues and mixed them with 200 μL of methanol containing specific markers. After centrifuging the mixture at 20000 g, we collected 200 μL of the liquid part and diluted it with 2% formic acid. Next, the extracts were further enriched in phytohormones by solid‐phase extraction using 96‐deep well filter plates (Agilent Technologies) packed with 50 mg of a strong cation exchange HR‐XC material (Macherey & Nagel) and eluted with 900 μL acetonitrile. This acetonitrile fraction was then dried under a nitrogen flow and resuspended in 300 μL 50% aqueous acetonitrile followed by analysis using advanced UHPLC–MS/MS technology.

### 2.8 Inulin and Small Sugar Analysis

Carbohydrate analysis was performed to determine the concentrations of free fructose, sucrose, glucose, the inulin content, and inulin mean degree of polymerization (mDP). Free sugars were extracted from 30 mg freeze‐dried root material in 800 μL phosphate buffer (20 mM, pH 7) at 85°C for 30 min using intermittent agitation. After centrifugation for 15 min at 20000 g, the supernatant was collected and the pellet was resuspended in 400 μL phosphate buffer, and the extraction procedure was repeated. Supernatants were pooled and samples were de‐ionised with a mixture of Q‐Sepharose and S‐Sepharose beads in phosphate buffer (20 mM, pH 7). Samples were incubated for 5 min while shaking. After centrifugation, the supernatant was collected and analysed. Free glucose, fructose, and sucrose concentrations were determined by ion chromatography using a Thermo Fischer ICS 5000 + DC system (Thermo Fischer Scientific) equipped with a Carbo Pac PA‐1 column (4–250 mm) and a 25 μL sample loop, as described by (Stoop et al. 2007) with some modifications. Monosaccharides were eluted from the column with a flow rate of 1 mL min^−1^ using a NaOH gradient: from 0–10 min, the concentration of NaOH in the eluent increased from 20 mM to 50 mM in a linear manner; from 10–20 min a linear gradient from 50 mM to 330 mM was applied, followed by an isocratic elution using 330 mM NaOH for 5 min. An ED40 electrochemical detector fitted with a pulsed amperometric cell was used. Quantification of the three sugars was achieved by comparison of peak areas to external glucose, fructose, and sucrose standards (Merck KGaA). The inulin content was analysed in hydrolysed extracts. Hydrolysis was performed by incubating 30 μL de‐ionised sample with 30 μL of 200 mM HCl for 2 h at 60°C. Glucose and fructose concentrations were determined by ion chromatography as described for free sugars. The inulin content was calculated as the total carbohydrate content from which the concentrations of free glucose, fructose, and sucrose were subtracted. The mDP was calculated as the sum of bound glucose and fructose, divided by the amount of bound glucose.

### 2.9 RNA Isolation and Sequencing

Root tissues from the O37 line at 16‐week of age were used for dissection of the different cell types from the root sections. Dissected tissue and harvested latex from three independent biological replicates were frozen in liquid nitrogen. The different cell types were ground to a fine powder in the presence of liquid nitrogen and transferred to a −80°C freezer. 100 mg of biomass was used to isolate total RNA with an RNAeasy kit (QIAGEN). We then analyzed the RNA with a NanoDrop and BioAnalyzer 2100 (Agilent Technologies Inc) to evaluate the RNA concentration and RNA integrity number (RIN > 7). The RNA was sequenced in 150 bp paired‐end mode using an Illumina NovoSeq 6000 (Novogene). The RNAseq data sets have been listed in NCBI (www.ncbi.nlm.nih.gov) as a BioProject with the accession number of PRJNA824299 and the transcriptome assembly is accessible under the accession GKBE00000000.1.

The gene expression pattern of different tissues from chicory roots was elucidated in various types of root cells, including latex, HD, and VC. Dissection of different tissues from chicory roots was carried out with the guidance of stained sections (Figure S2). For this aim, paired sections with a diameter of 2 mm were selected; one was stained by TBO to differentiate various cell types and the other was used for dissection. The NGS transcriptional analyses of different cell types were produced via RNA sequencing of the same material that was used for the LC–MS study as this provides the possibility of doing transcriptome and metabolome data co‐analysis.

### 2.10 Transcriptome Assembly, Functional Annotation and Gene Expression Analyses

The quality of raw reads was examined by FastQC v 0.11.5 (https://www.bioinformatics.babraham.ac.uk/projects/fastqc/). Trimmomatic v0.32 (Bolger et al. 2014) was used for cleaning up the paired‐end Illumina raw reads from low‐quality and sequencing adapters. A de novo approach using Trinity (Grabherr et al. 2011) was used for assembly of the high‐quality reads (default parameters). bowtie2 (Langmead and Salzberg 2012) and RSEM (Li and Dewey 2011) were used to map the trimmed reads on the Trinity assembly and estimate the abundance of the read count subsequently. We normalized read counts to derive gene expression values as transcript sequence per million of mapped reads (TPM). We performed all statistical analysis using EdgeR and used quantitative PCR to confirm the results of RNA‐seq data for specific genes.

Transcriptome annotation was carried out using in‐house BlastX (cut‐off *E*‐value of 10–5) against the following databases: Nr, NCBI non‐redundant database (January 12, 2025); TAIR, The Arabidopsis Information Resource (TAIR10); SwissProt, TrEMBL, UniProt Knowledgebase (UniProtKB) and KOG (eukaryotic orthologue groups); (Koonin et al. 2004). Gene ontology and KEGG (Kanehisa 2000) annotations were generated by Blast2GO (Conesa et al. 2005) using BLASTx hits mining the Nr database. GO functional classification was carried out using DAVID (Huang et al. 2008) KEGG Mapper (Kanehisa and Sato 2019) online tools.

### 2.11 Reverse Transcriptase Quantitative PCR (RT‐qPCR)

We used total RNA from different cell types prepared as in section 2.9 and treated it with 1 μg of DNase (RQ1, Promega) for reverse‐transcription by Superscript III (Life Technologies). The cDNA (100 ng) was amplified by the BioRad Real‐Time PCR System under the operation of the CFX manager software, using 0.5 μM of each primer in a 20 μL final volume. The real‐time conditions were as follows: 95°C for 5 min, 45 cycles at 95°C for 15 s, 65°C for 15 s, 30 s. Agarose gel electrophoresis of the PCR product along with melting curve analysis was used to determine primer specificity. Samples were harvested from three independent biological replicates of O37 lines 16‐week‐old root tissues, and three technical replicates were performed for each sample. The actin gene (Maroufi et al. 2010) was used as a reference in the CFX manager software to normalize expression levels of the target genes. The expression of a sample of genes with tissue‐specific expression was verified and shown in Figure S10 along with the list of primers used.

### 2.12 Statistical Analyses

We applied principal component analysis (PCA) to samples of different cell types normalized to fresh weight in metabolome and transcriptome studies to evaluate the distribution and grouping among samples. PCA was plotted in R 3.6.1 using mean‐centered and standardized data (unit variance scaled). Differential expression analysis was carried out by the edgeR package (Robinson et al. 2009).

A trimmed mean of M values (TMM) normalization was applied to all samples during transcriptome data analysis. A threshold of log_2_ fold change ratio (log_2_ FC) ≥ 1 was used to define significant gene expression differences after cutting off the data at false discovery rate (FDR) value ≤ 0.05 for all unigenes with more than or equal to 1 TPM.

Gene ontology (GO) terms and Kyoto Encyclopedia of Genes and Genomes (KEGG; www.genome.jp/kegg/pathway.html) pathway analysis were carried out to identify biological functions and pathway involvement of differentially expressed genes (DEGs). Gene expression differences with *p* ≤ 0.05 were considered statistically significant (Kanehisa 2000). The fold enrichment (FE) was calculated based on Huang et al. (2008) as the ratio of the proportions of the treatment to the background.

For the co‐analysis of the metabolome and transcriptome data we used the Color tool in KEGG Mapper by submitting the respective annotated features from the metabolomics and transcriptomics datasets (Kanehisa and Sato 2019). Since the pathway mapping files of *Cichorium intybus* have not been introduced in the KEGG, we have used *Lactuca sativa* for the mapping of the data to the pathways.

## 3 Results

Morphological and cytological analysis showed the existence of different tissues and compartments across the taproots, including latex (LX), hypodermis (HD), cortex (CX), phloem (PH), and vascular cylinder (VC; Figure S1). It is difficult to properly dissect the CX and PH, but it is possible to dissect the HD and VC without contamination from the other tissues. We also collected the latex (LX), which is the cytosolic compartment of laticifer cells, by cutting plant roots and shoot parts. Therefore, in this study, we have used three different root tissues (LX, HD, and VC) for targeted and untargeted metabolic profiling. In addition, we generated transcriptome data of LX, HD, and VC to determine differentially expressed genes in these tissues from 16‐week‐old plants.

### 3.1 Metabolome Profiling of STLs

#### 3.1.1 STLs Profiling of Different Root Cell Types

Although it is known that in latex‐producing Asteraceae species, the STLs mostly accumulate in the latex (Seo et al. 2009; Sessa et al. 2000), there is no clear picture of the distribution of STLs in chicory roots. To analyze the pattern of STLs distribution across the plant tissues, we measured the peak area of STLs in three different plant tissues using a targeted approach (Table S1). The major STLs of chicory are lactucin (L), 8‐deoxylactucin (DL), lactucopicrin (LC), lactucin‐15‐oxalate (LOx), 8‐deoxylactucin 15‐oxalate (DLOx), and lactucopicrin oxalate (LCOx; Figure 1).

**FIGURE 2:**
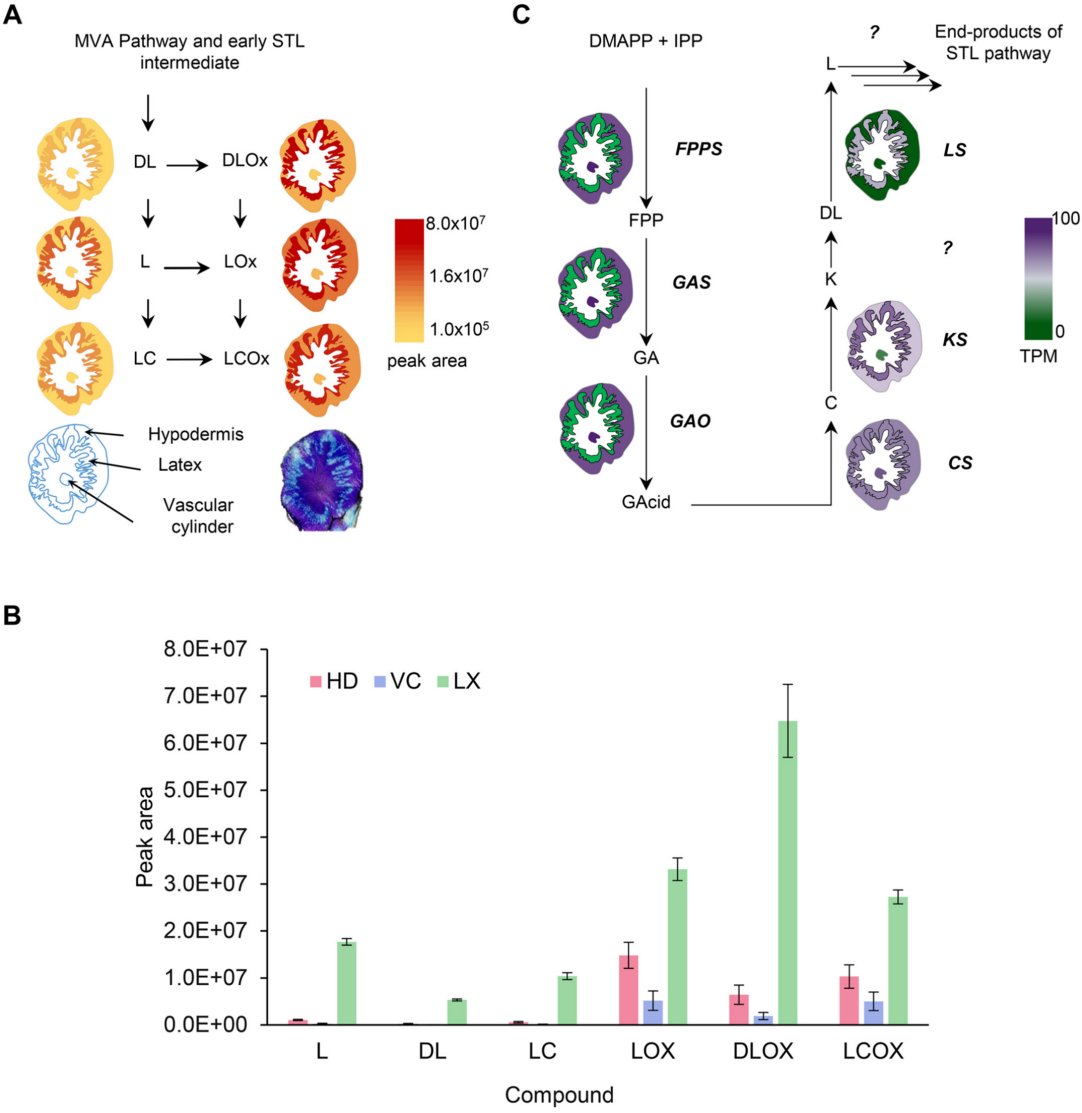
Profiling of sesquiterpene lactones (STLs) and STL‐biosynthesis pathway gene expression in chicory roots. (A) Distribution of the major STLs in different root tissues using targeted LC–MS positive mode. DL, deoxylactucin; DLOx, deoxylactucin‐oxalate; L, lactucin; LOx, lactucin‐oxalate; LC, lactucopicrin; LCOx, lactucopicrin‐oxalate. The color scale indicates the peak area of the measured compounds normalized to tissue fresh weight in logarithmic progression. (B) Graph of the peak areas of major STLs as measured by LC–MS in positive mode. Values are the average of three biological replicates. Error bars represent the standard error. (C) Expression pattern of the known steps of STLs biosynthesis across root tissues. Abbreviations: C, costunolide; *CS*, costunolide synthase; DL, deoxylactucin; DMAPP, dimethylallyl diphosphate; FPP, farnesyl diphosphate; *FPPS*, farnesyl diphosphate synthase; GA, germacrene A; GAcid, germacrenoic acid; *GAO*, germacrene A oxidase; *GAS*, germacrene A synthase; IPP, isopentenyl diphosphate; K, kauniolide; *KS*, kauniolide synthase; L, lactucin; *LS*, lactucin synthase. TPM: Transcripts per million.

The STLs were measured from root sections by collecting material from three different tissues: LX, HD and VC (Figure S1). In order to dissect different tissues from chicory root, it was necessary to distinguish them by staining of neighboring sections (Figure S1). Principal component analysis of STLs in the three different root tissues shows a clear separation of the sample types (Figure S2), with STLs in latex being significantly higher than in other tissues (Figure 2A,B). By contrast, the VC has the lowest concentrations of STLs. The oxalate forms of STLs are more abundant than non‐oxalates in all cell types (Figure 2A,B). Thus, our analysis of STLs in chicory roots confirms that they are largely stored in the latex, as shown in other related species.

### 3.2 Untargeted Metabolome Profiling of Chicory Root Tissues

**FIGURE 3:**
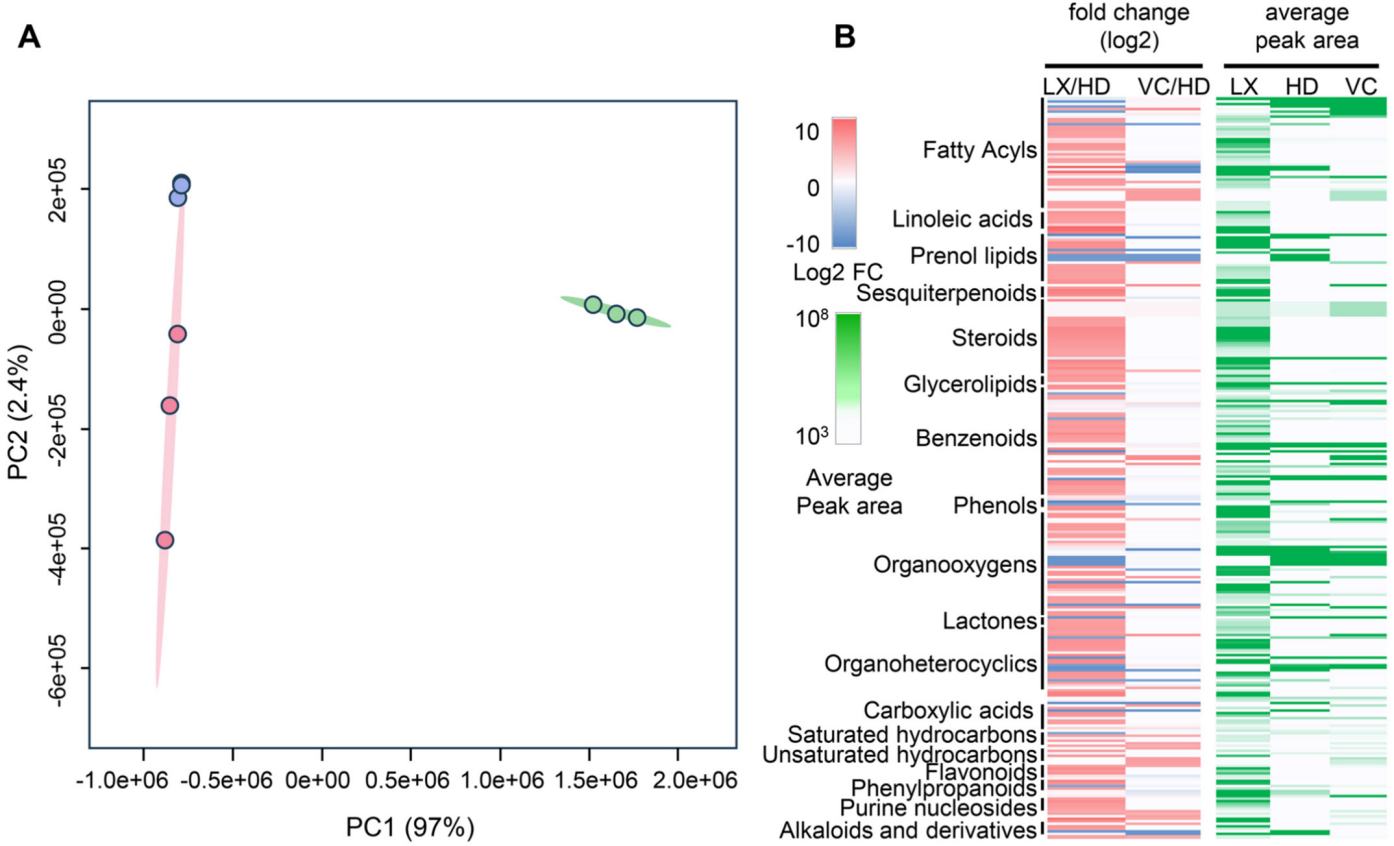
Metabolome profiling of chicory roots. (A) Principal component analysis of various biological repeats across three root tissues based on the peak area from metabolite profiling using untargeted profiling (LC–MS‐neg). (B) Abundance (right side) and fold change relative to HD (left side) of classes of metabolites across three root tissues. HD, hypodermis; LX, latex; VC, vascular cylinder.

To explore the metabolite distribution pattern across different cell types of chicory roots, we measured metabolites extracted from two tissues (HD and VC) and LX using exhaustive LC–MS and GC–MS profiling (Figure 3). Untargeted LC–QToF‐MS/MS was performed using both negative (LC–MS‐Neg) and positive (LC–MS‐Pos) ionization modes for medium polar metabolites (mpm). In addition, we used an untargeted profiling approach for hydrophilic metabolites (LC–MS‐hpm) (Balcke et al. 2017). GC–MS was done with hexane extracts. The untargeted metabolite analysis aimed to reveal metabolic processes in chicory roots, focusing on inulin and STLs biosynthesis, compartmentalization, and trafficking. Through GC–MS and LC–MS techniques, 21,437 features were identified, with 135 showing differential abundance across cell types, shedding light on STL and inulin distribution and associated metabolic pathways (Figure S3).

Principal component analysis (PCA) of the samples based on metabolome data using different MS approaches again showed clear separation of the samples according to tissue type, for which the LC–MS–neg dataset constitutes a representative example (Figure 3A). A total number of 21,437 features were identified from the metabolome profiling data of chicory root tissues using five different methods. LC–MS‐hpm with 12,954 and GC–MS with 1657 features produced the largest and smallest datasets, respectively. Annotation of the different datasets from various cell types identified a total number of 878 potential metabolites. The highest and lowest rates of annotation were for GC–MS and LC–MS‐Pos, respectively, while LC–MS‐hpm produced the largest dataset (Figure S3). In the GC–MS, LC–MS‐hpm, LC–MS‐Pos, and LC–MS‐Neg measurements, the most abundant compounds across all tissues are present in the LX samples (Figure S3). Based on the ClassyFire annotation (Djoumbou Feunang et al. 2016), these compounds belong to lipids, fatty acids, steroids, sesquiterpene lactones, and their derivatives. Also, across all tissues, the most abundant compounds in LC–MS‐Neg measurements are present in the VC (Figure S3). Based on the ClassyFire annotation, these compounds mainly belong to the subclass of alcohols and polyols, amino acids and peptides, fatty acids, and conjugates (Figure S3 and Table S2).

In addition to STLs, other mevalonate pathway‐derived metabolites such as squalene, triterpenes, and sterols accumulate in the latex, similar to what was shown in Russian dandelion (*Taraxacum koksaghyz*) (Benninghaus et al. 2020). Sterols and fatty acids are involved in structural integrity and energy storage and occur in LX in association with rubber (Schulze et al. 2011). These molecules are present in higher amounts in the LX of chicory roots compared to other tissues (Figure 3B and Table S2).

Pathway enrichment analysis of the annotated compounds indicates that the majority of the compounds map to the biosynthesis of secondary metabolites (*n* = 58). *cis*‐1,4‐polyisoprene is a major component of natural rubber, and the rest consists of related proteins, carbohydrates, lipids, sterols, and terpenoids (Schulze et al. 2011). Consistent with this, pathways related to the biosynthesis of unsaturated fatty acids (19 features) and fatty acid biosynthesis (11 features) were enriched in LX. In total, the LX contains most of the compounds (75 features) mapped to metabolic pathways, and VC holds the lowest numbers (9; Table S4).

As shown in Figure S3, most of the compounds could not be annotated using the MS‐Dial or Xcalibur software programs. An enriched annotation of the metabolome is a necessary step for understanding it. Therefore, to associate the unknown compounds with known classes, we used MetFamily, a software that clusters MS features based on the similarity of their fragmentation patterns (Treutler et al. 2016). For instance, we found, as expected, that the most abundant compounds detected in the latex by MS/MS in the negative mode belong to the group of STLs (Figure S4 and Table S4).

### 3.3 Transcriptome Profiling

The percentage of mapped reads is an important mapping quality statistic since it is a global indicator of overall sequencing accuracy and the presence of contaminating DNA (Conesa et al. 2016). For instance, depending on the read mapper utilized, 70%–90% of normal RNA‐seq reads will map onto the human genome (Dobin et al. 2013). Despite the availability of the genome for *Cichorium intybus* , Cultivar: Punajuju (ASM2352571v1), we decided to use de novo transcriptome assembly because of the lower mapping rate of O37 RNA sequencing reads on the genome of the Punajuju (<65%) in comparison to the de novo assembled transcriptome (>95%; Figure. S5) and also due to the lack of publicly available information about the Punajuju variety.

#### 3.3.1 Tissue‐Specific Gene Expression Based on RNA‐Seq

We performed transcriptome analysis of the same root tissues used for metabolome profiling, namely HD, VC and LX (Figure. S2). RNA samples were processed for RNA sequencing using Illumina sequencing. We performed differential gene expression analysis for the three tissues. The biological replicates of each of these tissues cluster tightly together and each cluster has a distinct expression pattern, supporting a reliable preparation of the tissues. LX has the highest number of tissue‐specific genes (3784), followed by HD (1153) and VC (699). HD and VC have more commonly expressed genes (1736), followed by LX and HD (857) and LX and VC (447; Figure S6). Mapping of annotated transcripts to pathways using KEGG mapper (Kanehisa 2000) showed that the biosynthesis of secondary metabolites, hormone signal transduction, plant‐pathogen interaction and phenylpropanoid biosynthesis were the most enriched processes across different cell types (Table S4).

#### 3.3.2 Biosynthesis of STLs Occurs Across Several Tissues

**FIGURE 4:**
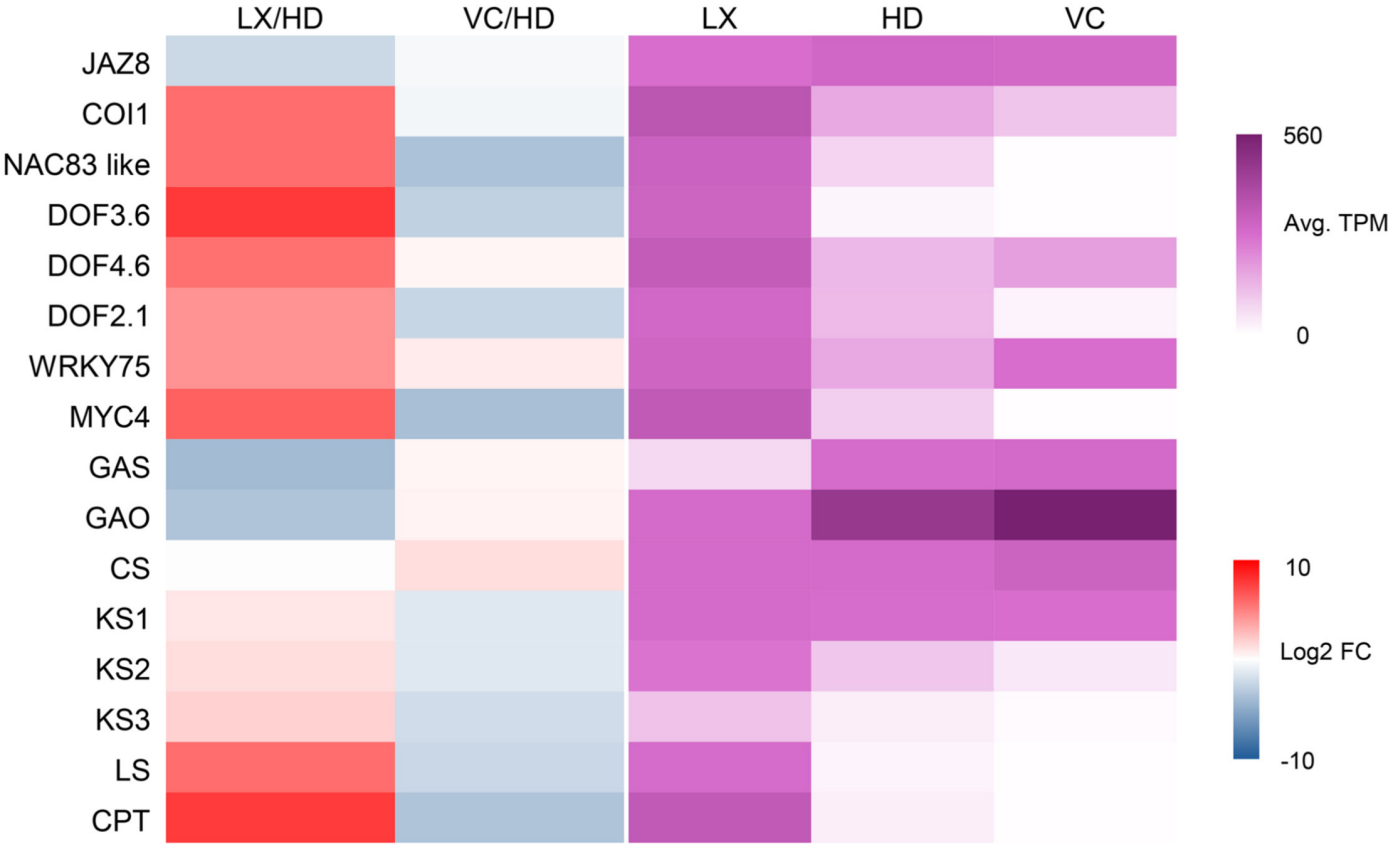
Expression data of selected differentially expressed genes. Left panel: Log2 fold change of latex to hypodermis (LX/HD) and vascular cylinder to hypodermis (VC/HD). Right panel: Gene expression values in transcripts per million (TPM). *COI1*, jasmonate receptor; *CPT*, poly *cis*‐prenyltransferase; *CS*, costunolide synthase; *DOF2.1, DOF3.6*, *DOF4.6*, *MYC4, NAC83 like*, *WRKY75*: Transcription factors; *GAO*, germacrene A oxidase; *GAS*, germacrene A synthase; *JAZ8*, jasmonate repressor; *KS1‐3*, kauniolide synthase; *LS*, lactucin synthase.

To gain further insight in the distribution of STL biosynthesis across root tissues, we collected gene expression data for the known genes of the pathway, namely farnesyl diphosphate synthase (*FPPS*), germacrene A synthase (*GAS*, Bouwmeester et al. 2002), germacrene A oxidase (*GAO*; Nguyen et al. 2010), costunolide synthase (*COS*; Ikezawa et al. 2011), kauniolide synthase (*KLS*; Cankar et al. 2022), and lactucin synthase (*LCS*; Cankar et al. 2023). Our transcriptome data show that genes involved in the early steps of STLs biosynthesis, including *FPPS*, *GAS*, and *GAO*, are expressed in HD and VC. However, the next step, *COS*, is expressed at similar levels in all tissues, while the known downstream steps (*KLS* and *LCS*) are preferentially expressed in the latex compared to HD and VC (Figure 2C; Figure 4).

#### 3.3.3 Differential Expression of Transporters Across Chicory Root Tissues

The compartmentalization of STL biosynthesis gene expression would necessitate the transport of STL pathway intermediates between root tissues. Indeed, we observe striking differential expression of distinct classes of ABC transporters. In the LX, ABCB/E/F are highly upregulated, while ABCC/G are mainly expressed outside of the LX (Figure. S5; Table S5). High expression of the ABCG/C outside of the LX might be an indication of their role in exporting metabolites and precursors into the LX. ABCG is the largest subfamily of ABC transporters, which mainly play roles in the export of metabolites (Banasiak et al. 2021; Natarajan et al. 2020; Philippe et al. 2022; Wu et al. 2014) such as cutin precursors, cutin monomers, wax, cuticular lipids, alkanes, suberin (Xin and Herburger 2021), lipids (Banasiak et al. 2021), diterpenoids (Jasinski et al. 2001), and toxic metabolites (Goldstone 2008). In contrast, ABCB transporters are involved in the import of metabolites such as auxin (Paterlini 2020), secondary metabolites, and other substances (Xie et al. 2020).

#### 3.3.4 Genes Involved in Rubber and Triterpenoid Biosynthesis Are Overexpressed in Latex

In Russian dandelion, which is related to chicory, in addition to STLs the latex contains triterpenoids and natural rubber, whose biosynthesis also requires isoprenyl precursors from the cytosolic mevalonate pathway. In the rubber tree ( *Hevea brasiliensis* ) and Russian dandelion ( *Taraxacum kok‐saghyz* ), natural rubber biosynthesis takes place in the latex (Cherian et al. 2019). The biosynthesis of *cis*‐1,4‐polyisoprene takes place on the surface of rubber particles, with *cis*‐polyprenyl transferases (CPTs) being the major enzymatic activity (Cherian et al. 2019). A phylogenetic tree of CPT homologs present in chicory indicates that there are genes orthologous to known rubber CPTs from the related *Taraxacum* species (Figure. S8). CPTs alone cannot synthesize long chains of *cis*‐1,4‐polyisoprene but require the presence of additional proteins present in the rubber particles, including small rubber particle proteins (SRPP). As expected, genes encoding CPTs and CPT‐binding proteins (Epping et al. 2015; Qu et al. 2015) are specifically upregulated in the LX (Figure S7; Table S5). Also, higher expression of squalene synthase and squalene epoxidase in the latex (Table S5) is consistent with the accumulation of triterpenoids in the latex, as is the case in the related *Taraxacum* spp. (Pütter et al. 2017). Furthermore, genes encoding enzymes of the mevalonate pathway are strongly upregulated in the LX, consistent with its role in supplying precursors for the synthesis of natural rubber and triterpenoids (Figure S7; Table S5).

#### 3.3.5 Gene Expression for Inulin Metabolism and Distribution of Inulin and Related Sugars in Chicory Roots

**FIGURE 5:**
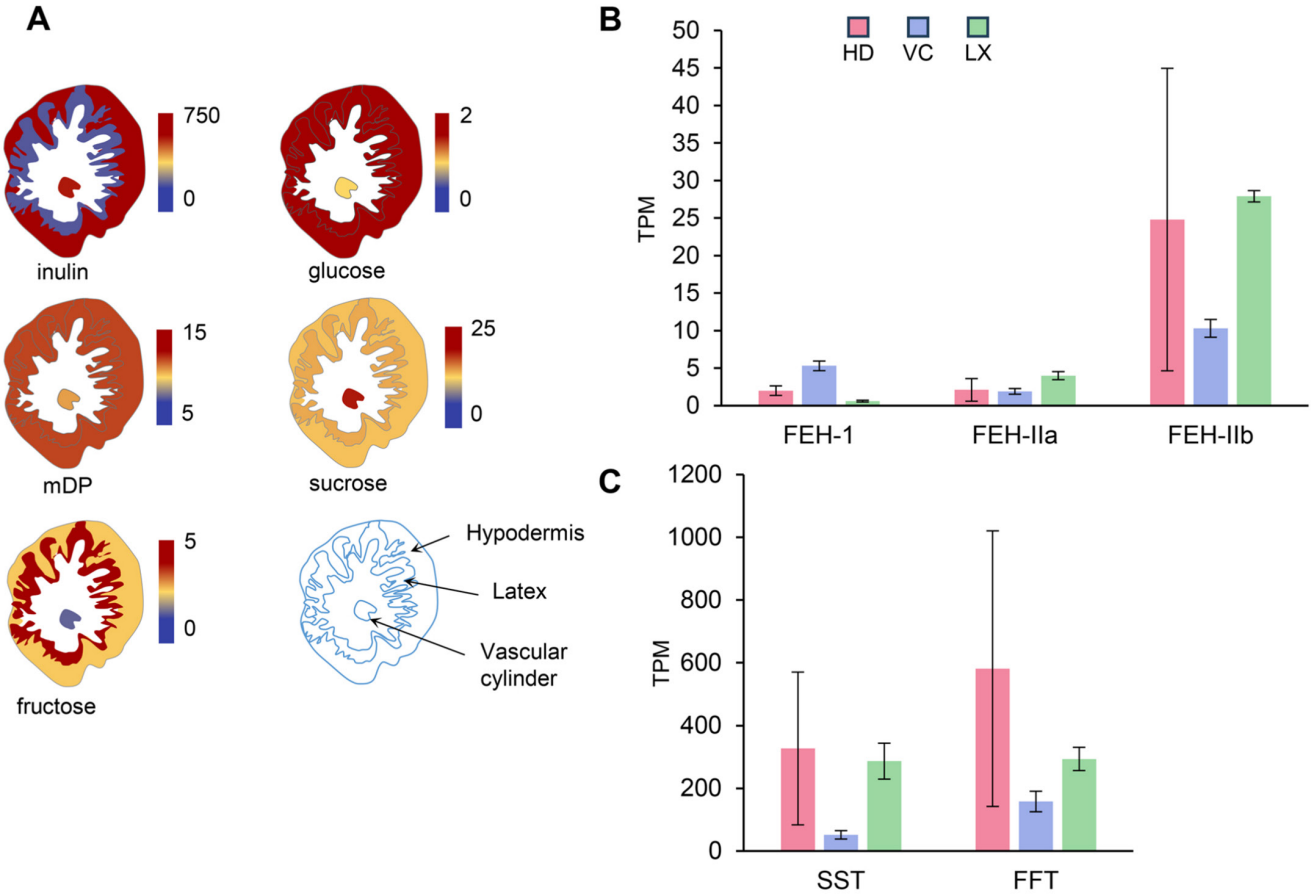
Inulin metabolism in chicory roots. (A) Content of inulin and related sugars. mDP: Mean degree of polymerization of inulin. Values are mg g^−1^ dry weight except for mDP. The data are available in Table S6. (B) Inulin metabolism gene expression in chicory roots. FEH, fructan exohydrolase; FFT: Fructan:Fructan 1‐fructosyltransferase; SST: Sucrose:Sucrose 1‐fructosyltransferase. TPM, average of transcripts per million from three biological replicates ± standard error.

The highest concentration of inulin is in HD and VC, whereas LX has the lowest content (Figure 5 and Table S6). Interestingly, sucrose, the building block of inulin, showed different distributions. Sucrose was most abundant in the vascular cylinder, with decreasing concentrations in the LX and HD (Figure 5). Glucose and fructose levels are relatively low compared to sucrose and especially low compared to inulin levels. Glucose is produced in the first step of inulin synthesis when sucrose–sucrose 1‐fructosyltransferase (SST) catalyzes the synthesis of the shortest inulin from two sucrose molecules. Fructose is produced when inulin is broken down by fructan exohydrolase (FEH) activity. Glucose was more abundant in the HD whereas fructose, surprisingly, was most abundant in the LX. Thus, fructose, which is a major ingredient of inulin, and released when inulin is degraded by FEH, is highest in latex which might point to breakdown of inulin in the LX. The genes for inulin biosynthesis, respectively encoding SST and FFT, are most strongly expressed in the HD (Figure 5). Nonetheless, these genes are also expressed relatively high in the LX and significantly less in the VC although inulin levels in VC are comparable to HD. This suggests that although expression of inulin biosynthesis genes seems to take place in LX, it might not lead to inulin biosynthesis enzyme activity in LX (Figure 5). However, the low levels of inulin in the LX combined with high levels of fructose suggest that inulin in the LX is rapidly degraded or exported to neighboring tissues. Interestingly, ABC transporters have also been shown to be responsible for the transport of inulin by bacteria (Tsujikawa et al. 2021). Individual ABCG transporters that are overexpressed in LX (Figure S7) constitute potential candidates for the export of inulin out of the laticifers. The expression of genes involved in the degradation of inulin encoding isoforms of fructan exohydrolase (FEH‐I, FEH‐IIa and FEH‐IIb) is rather low but for FEH‐IIa highest in LX and for FEH‐IIa and b highest in HD, and most likely they play a role in the low inulin level found in latex.

#### 3.3.6 Hormone Profiling Shows Increased Jasmonates and Abscisic Acid Levels in Latex

**FIGURE 6:**
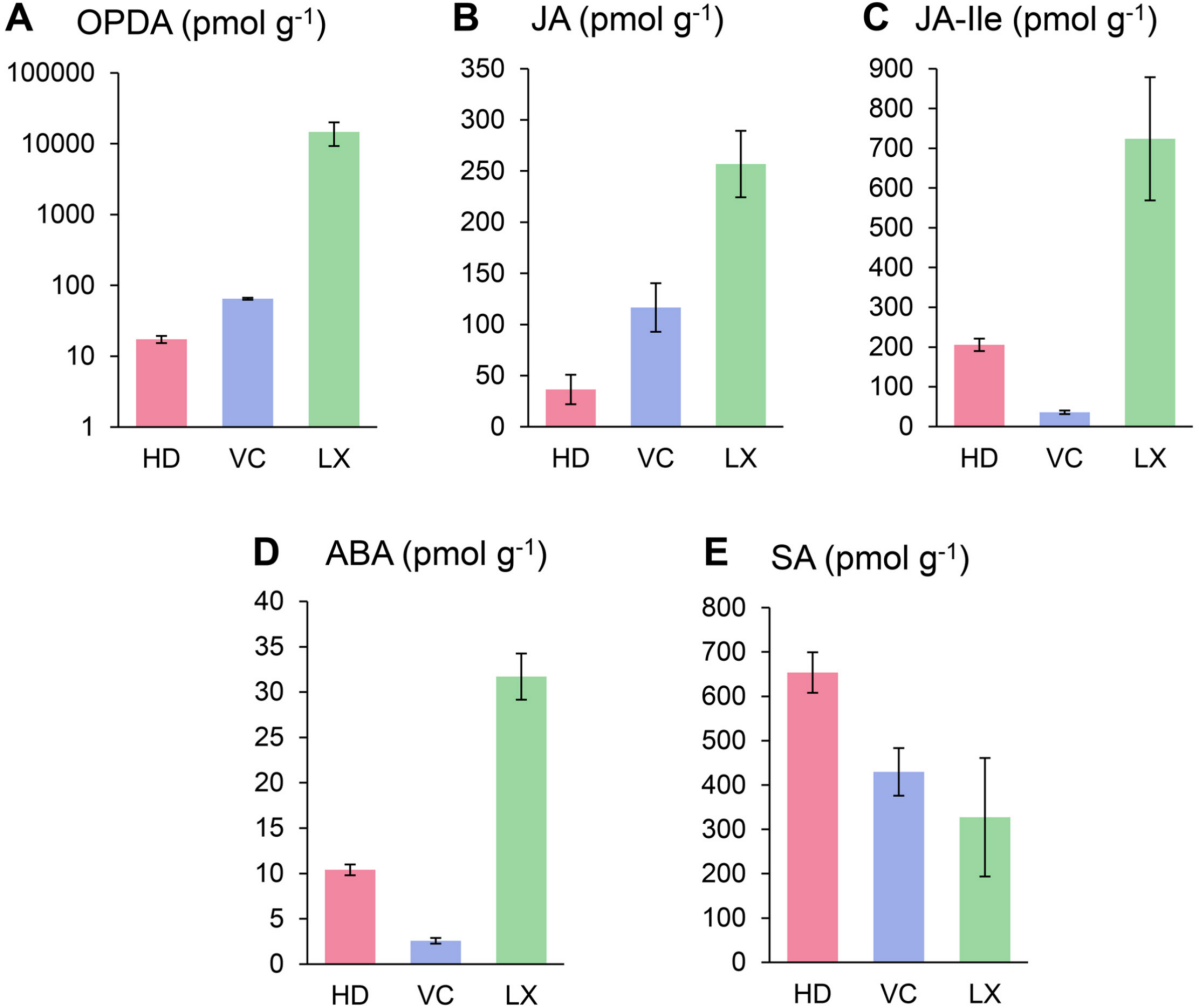
Concentration of different plant hormones in chicory root tissues. Concentrations are in pmol g^−1^ fresh weight. (A) OPDA: 12‐oxophytodienoic acid. (B) JA: Jasmonic acid. (C) JA‐Ile: Jasmonate isoleucine; (D) ABA: Abscisic acid. (E) SA: Salicylic acid. HD: Hypodermis; LX: Latex; VC: Vascular cylinder. The data are based on measurements from three biological replicates. The error bars indicate the standard error range. Number of biological replicates = 3.

There is little information available to date on phytohormone levels associated with laticifers in plants. We thus measured several phytohormones in LX, HD and VC with a focus on jasmonate (JA) related metabolites. Phytohormone levels for JA, OPDA, JA‐isoleucine and abscisic acid (ABA) were significantly higher in LX relative to other tissues, while SA levels were not significantly different between tissues (Figure 6). OPDA levels were particularly high in LX, reaching levels of several thousand pmol g^−1^ FW. It has been known that Me‐JA, OPDA and JA are involved in the modulation of the latex in the rubber trees (Duan et al. 2010; Hao 2000; Laosombut et al. 2016). However, this was done either by supplying exogenous jasmonates or upon wounding of the plants (Hao 2000).

#### 3.3.7 Other Aspects: Expression of Ubiquitination‐Related and Aquaporin Genes

Analysis of our transcriptome data indicates the upregulation of several elements of ubiquitin‐mediated protein degradation and protein degradation‐related signaling pathways (Table S4). Protein turnover is necessary for tissues with high metabolic activity (Quigg and Beardall 2003; Vierstra 1993), which is the case in laticifers (Tang et al. 2013; Tungngoen et al. 2009). Moreover, upregulation of six aquaporins was also observed in LX (Table S5). The aquaporin gene (HbPIP2;1, T11M8–2) was suggested to be involved in regulating the water transfer between the laticifers and surrounding tissues and its expression has been positively correlated with ethylene stimulation of latex yield (Tungngoen et al. 2009). Also, it has been suggested that upregulation of aquaporins is involved in the enhanced latex regeneration (Tungngoen et al. 2009).

#### 3.3.8 Integrative Analysis of Transcriptome and Metabolome Data

Integrative analysis of metabolome and transcriptome data would provide a comprehensive picture of the regulatory pathways and uncover processes that are not obvious through analysis of the individual omics data (Sharma et al. 2020). We thus performed co‐analysis of the transcriptome and metabolome data by mapping them to the KEGG pathways (Table S4 and Figure S6). Because the annotation of many metabolites is tentative and many metabolic features could not be annotated, this integrative analysis should be taken with caution.

Not surprisingly, metabolic pathways and the biosynthesis of secondary metabolites are strongly represented in LX (Table S4 and Figure S6). Noteworthy was the overrepresentation of vesicular transport and endocytosis in LX. This may be connected to the abundant metabolic pathways that occur in the laticifers, particularly rubber biosynthesis, and may also reflect the fact that the laticifers constitute a sink tissue. Furthermore, the highest pathway enrichment in LX was for the synthesis and degradation of ketone bodies. Ketone bodies are degradation products of lipids, whose role in animals is to serve as a circulating energy source during calorie restriction (Cahill Jr. 2006). They also have a signaling function in maintaining energy balance in animal cells (Newman and Verdin 2014). In the context of chicory roots, ketone bodies could represent an alternative energy source when sugars like fructose and glucose are limiting due to their use in inulin biosynthesis. Finally, the MAPK signaling pathway was also overrepresented in LX. Generally, MAPK signaling is involved in stress or hormonal responses (Taj et al. 2010). Both could play a role in LX, either in the development or in the metabolic status of laticifer cells.

In the HD, we noted a strong enrichment in autophagy (Table S4). In Euphorbia, autophagy plays a role in the development of non‐articulated laticifers through the fusion of autophagosomes and lysosome‐like vesicles (Zhang et al. 2018). Since laticifers emerge in the HD, this would also suggest a role for autophagy in the development of laticifers in chicory. The Glycosylphosphatidylinositol (GPI)‐anchor biosynthesis (GPI‐AP) pathway is one of the most enriched categories in both omics datasets in the HD (Table S4). GPI‐AP is involved in facilitating trafficking routes and is anchored at the cell surface (Maeda and Kinoshita 2011). This is also consistent with the high regulation of the ABCG/C in the HD (Figure S7), which may be relevant for the transfer of metabolites to the LX. We note also the enrichment in several lipid biosynthesis pathways (sphingolipid, glycerolipid and unsaturated fatty acid) in HD. This again may have to do with the transfer of metabolites to the laticifer but also possibly with the storage of inulin in the vacuole (Darwen and John 1989).

Finally, there is a strong enrichment of the categories fructose and mannose metabolism in all tissues (LX, HD and VC). This is most likely due to the biosynthesis of inulin, which is a major process in chicory roots.

## 4 Discussion

In this work, we generated a comprehensive dataset of metabolomics and transcriptomics for different tissues of the chicory root. Chicory is a specialized crop for the high‐scale production of inulin, which accumulates in large quantities in the taproot. In addition, chicory produces a milky latex that contains high amounts of sesquiterpene lactones (STLs) as well as *cis*‐1,4‐polyisoprene. The presence of the latex represents a problem for inulin processing but also a potential opportunity for the production of STLs, some of which have potential pharmacological applications (Häkkinen et al. 2021; Matos et al. 2020; Peña‐Espinoza et al. 2015; Peña‐Espinoza et al. 2020). Furthermore, Russian dandelion (*Taraxacum koksaghyz*) and guayule ( *Parthenium argentatum* ) produce a milky latex whose *cis‐1,4‐polyisoprene has* properties that are similar to those of the rubber tree ( *Hevea brasiliensis* ) and constitute potential replacements for natural rubber but with a better allergenic profile. Thus, generating tissue‐specific transcriptomics and metabolomics should provide insights both into the production of inulin but also on the development of the laticifers as well as the biosynthesis of compounds accumulating in the latex. Currently, such a comprehensive and well documented study for chicory and related species is not available. The most salient results of our multiomics dataset are discussed below.

### 4.1 Biosynthesis of Sesquiterpene Lactones Occurs Across Several Tissues

STLs are the major bitter compounds associated with chicory taste and are one of the reasons for using chicory as a coffee replacement. The most abundant STLs accumulate in the LX, particularly deoxylactucin, lactucin and lactucopicrin and the oxalate derivatives thereof, which collectively represent the end‐products of the pathway (Figure 2). All STL end‐products are most abundant in the LX, but they can also be detected in the HD and in the VC, albeit at much lower levels (Figure 2). This can be attributed to leakage from the latex or possibly transport between those tissues. Interestingly, we were unable to detect intermediates such as costunolide, suggesting a complete conversion to the late products of the pathway. Only the knock‐out of the genes encoding kauniolide synthase (KS) allowed the detection of costunolide, but mostly in the form of cysteine or glutathione conjugates (Cankar et al. 2022). However, the tissue localization of costunolide and associated derivatives was not determined. Therefore, this raises the question of the distribution of the STL pathway enzymes and intermediates across the chicory root tissues. Insights into pathway distribution can come from tissue specific gene expression data. All biosynthesis steps until kauniolide are known, including germacrene A synthase (GAS), germacrene A oxidase (GAO), costunolide synthase (CS) and kauniolide synthase (KS; Cankar et al. 2022). The only step known beyond kauniolide is lactucin synthase (LS; Cankar et al. 2023). Our gene expression data indicate that up to costunolide, the biosynthesis takes place outside the LX and that for the known steps beyond costunolide, i.e., KS and LS, the biosynthesis is localized in the LX (Figure 4). These data are consistent with those of previous studies, where the expression of genes for GAS, GAO and CS investigated by RT‐qPCR was shown to be low in the LX (Kwon et al. 2022) and promoter:reporter gene fusions confirmed the expression of GAS in the root cortex (Bogdanović et al. 2019; Kwon et al. 2022). Still missing are oxidases for positions 2 and 15, an acyl‐transferase for the conjugation of oxalate at position 15 and a transferase for the transfer of hydroxyphenyl acetate at position 8 for lactucopicrin. The oxidases are likely cytochrome P450 oxygenases of the CYP71 family, as is the case for most oxidases identified so far in STL biosynthesis from the Asteraceae (Frey et al. 2018; Frey et al. 2019; Frey et al. 2020; Liu et al. 2014; Liu et al. 2018; Teoh et al. 2006). A phylogenetic tree of CYP‐encoding genes differentially expressed in latex (Figure S9) reveals that indeed most belong to the CYP71 family and are therefore likely candidates for the missing steps in the pathway. If confirmed, this would suggest that most steps after costunolide are indeed localized in the latex. Altogether, the combination of metabolic profiling and gene expression data indicates that intermediates up to germacrenoic acid (GA) and costunolide (C) are synthesized primarily outside the latex, and that germacrenoic acid and costunolide are then transported to the laticifer cells and there further converted to the end‐products of the pathway.

Thus, although transport of other intermediates cannot be excluded, it is probable that GA and C are the major intermediates transferred from surrounding tissues to the latificer cells.

The distribution of pathways for specialized metabolites across several tissues is not uncommon in plants. For example, in the early enzymatic steps of the opium poppy ( *Papaver somniferum* ), benzylisoquinoline alkaloids that accumulate in the latex occur in the sieve elements of the phloem and the corresponding genes are transcribed in the companion cells (Onoyovwe et al. 2013; Ozber and Facchini 2022). Similarly to the situation in chicory, the late steps of benzylisoquinoline alkaloid biosynthesis do occur in the latex (Chen et al. 2018; Onoyovwe et al. 2013). Additionally, some steps in benzylisoquinoline alkaloids biosynthesis do occur in both tissues, indicating that several intermediates may be transported from sieve elements to the latex (Onoyovwe et al. 2013).

### 4.2 Implications for Transport

Consistent with the expression of the STL biosynthesis pathway across several root tissues, distinct classes of ABC transporters show differential expressions between LX and outside of LX. In opium poppy there is an indication that the transfer of intermediates from sieve elements to laticifers is apoplastic rather than symplastic. The recent identification of a small group of purine‐permeases involved in the import of benzylisoquinoline alkaloids into the laticifer cells confirms this hypothesis (Dastmalchi et al. 2019). Whether such permeases are also involved in the import of GA or C into laticifers is questionable, however, because sesquiterpenoids are structurally very different from alkaloids. Instead, we noted differential expression for a number of ABC transporters (Table S5; Figure S7). Fittingly, a number of ABCG transporters, which are known to be exporters, are overexpressed in the HD, while ABCB transporters, which in general are importers, are overexpressed in the LX (Table S5). The latter therefore represent potential candidates in the import of GA or C into the laticifers, while the former are candidates for the export of GA or C from the HD. Validation of the function of these candidate transporters could be done by creating knock‐out lines by gene editing. However, the redundancy within the ABC transporter family, as visible from the number of transporters differentially expressed in chicory, may require inactivating several genes to see a noticeable effect.

### 4.3 Implications of the Distribution of Inulin and Sugars in the Root Tissues

Analysis of inulin content across the root tissues clearly shows that it accumulates throughout the root, except in the LX (Figure 5). Therefore, sucrose, which is the substrate for inulin biosynthesis and is imported from the leaf, would have to be transported from the phloem across the laticifers. The high concentration of fructose in the LX and the relatively high expression of inulin degradation genes in the LX suggest that some inulin is degraded in the LX, but that both inulin and fructose might also be exported to neighboring tissues. However, the degree of polymerization of inulin in the LX is similar to that of the HD (Figure 5). This suggests that inulin is not extensively broken down in the LX but exported, or that shorter chain inulins are exported. One way to clarify this issue would be by identifying the trafficking mechanisms of inulin between tissues. In plants these are not known, but bacterial ABC transporters were shown to be involved in the import of inulin (Tsujikawa et al. 2021), indicating that again ABC transporters may also be involved in the trafficking of inulin. Further studies will be needed to investigate the dynamics of inulin biosynthesis and transport in chicory roots during development.

### 4.4 Composition of the Latex

The latex is a complex mixture of multiple compounds. As mentioned above, it contains small molecules in quite large amounts, such as sesquiterpene lactones and triterpenoids. However, a typical component of latex is rubber (poly‐*cis*‐1,4‐isoprene), which plays an important role in sealing wounds via coagulation mediated by oxidases (Wahler et al. 2009). To the best of our knowledge, there is no report available on the presence and quality of rubber in chicory. As related Asteraceae species like Russian dandelion, guayule, or lettuce represent alternative sources for natural rubber to replace the rubber tree, it is likely that chicory also produces rubber. This is confirmed by the strong overexpression in the LX of genes typically associated with rubber production, namely *cis*‐polyprenyl transferase (CPT), rubber elongation factor, and small rubber particle protein (Figure 4; Table S5). However, the length and amount of rubber produced by chicory are not known, and it would be worth investigating this further to evaluate the potential of chicory as a rubber producer. In contrast to Russian dandelion, the cultivation of chicory is well established in the north of Europe. It is also a highly productive crop with large taproots, although it has been bred for the production of inulin. Increased latex production could also be a breeding target, which may be more accessible nowadays with the availability of genome editing technologies. This will require deeper insights into the molecular processes that regulate laticifer development and the biosynthesis of rubber. One aspect of this is the hormonal regulation of development and biosynthetic processes.

### 4.5 The Potential Role of Phytohormones and Transcription Factors in Laticifer Formation and Latex Production

In *H. brasiliensis*, wounding and application of JA, methyl jasmonate (MeJA), linolenic acid and coronatine result in stimulation of laticifer formation (Hao 2000; Loh et al. 2019; Tian et al. 2015; Zhang et al. 2016). It has been suggested that JA promotes the differentiation of laticifer cells (Hao 2000; Tan et al. 2014) via the enrolment of JAZ, NINJA, TOPLESS (TPL), 28S proteasome, *SKP1*, *MYC1*, *MYC2*, and *EREBP1* in mature rubber trees (Chen et al. 2011; Yang et al. 2011; Zhao et al. 2011). Also, expression of *COI1*, *MYB*, *ARF8* and *HB8* is correlated with MeJA‐mediated differentiation of the laticifers (Laosombut et al. 2016). However, all these data are based on artificial exposure to jasmonates, either by wounding or by applying exogenous JA or related substances to rubber trees. Thus, it is still unknown whether jasmonate signaling is involved in the developmental program and differentiation of laticifers. Several pieces of data in this work point to such a role for jasmonates in chicory. A role for jasmonates and jasmonate signaling in laticifer development is supported both by high levels of jasmonates in the LX compared to other tissues (Figure 6) and by the upregulation of a gene encoding the jasmonate receptor, COI1, in the LX (Figure 4). Additionally, in our transcriptomics data, several JA‐related genes are upregulated in the LX (Table S4).

Furthermore, in the latex, the majority of JAZ‐encoding genes, which are transcriptional repressors targeted by jasmonate signalling, exhibit slight upregulation or no differential expression (Table S3). Interestingly, a *JAZ8*‐like gene is downregulated in the LX (Figure 4, Table S5). Unlike other JAZs, JAZ8 does not possess a canonical degron (Shyu et al. 2012). The JAZ degron is a conserved LPIAR motif that is responsible for the proteolysis of the JAZ proteins when they form a complex with COI1 in the presence of JA‐Ile (Sheard et al. 2010). Consequently, JAZ8 suppresses JA‐regulated responses when expressed ectopically in *Arabidopsis thaliana* (Shyu et al. 2012). Therefore, it is possible that the chicory JAZ8, which is downregulated specifically in the latex, plays a role as a negative regulator in the process of laticifer development and differentiation and thereby restricts the expansion of laticifers in neighboring tissues.

Finally, several genes encoding transcription factors including a NAC, several DOFs, a MYC, and a WRKY are strongly upregulated in the LX (Figure 4, Table S5). In the rubber tree, *hbNAC1* is induced by wounding and dehydration and shown to activate the expression of an *SRPP* gene (Cao et al. 2017). Here also, gene editing could provide insights in the role of these transcription factors in laticifer development, although we can expect functional redundancy here as well.

## 5 Conclusions

This study provides new insights into the metabolism and gene expression of chicory roots and reveals a complex interplay of metabolic and developmental processes. From this dataset, it is possible to predict candidate genes involved in the biosynthesis of latex components (STLs, rubber and triterpenoids), in their transport and regulation, in laticifer development, and inulin metabolism and transport. Because chicory does not benefit from extensive genetics, in particular due to self‐incompatibility, the candidate genes identified by omics approaches can be tested with novel genome editing techniques, thereby bypassing the need for lengthy breeding approaches. This should lead to rapid improvements of the chicory crop, not only for inulin production but also for the development of new products present in the latex.

## Author Contributions

A.T. and K.V. designed the experiments. K.V. produced and analyzed the transcriptome and metabolome data. G.U.B. provided supervision for the production and analysis of the metabolome data. B.A. processed and analyzed the transcriptome data. J.C.H. and I.M.M. analyzed the inulin and sugar content. A.T. and K.B. wrote the manuscript, which was proofread and approved by all authors.

## Funding

This work was supported by the European Commission (760891).

## Disclosure

Significance Statement: A combination of transcriptomics, targeted and untargeted metabolomics of different tissues of chicory roots was generated. These data constitute a resource basis for the investigation of various processes taking place in chicory taproots, including sesquiterpene lactone biosynthesis, laticifer development, and inulin biosynthesis and trafficking.

## Conflicts of Interest

The authors declare no conflicts of interest.

## Supporting information


**Figure S1:** Chicory root tissue visualization.
**Figure S2:** Principal component analysis of STLs in chicory roots.
**Figure S3:** Overview of the metabolomics data from chicory roots.
**Figure S4:** Excerpt of a MetFamily output focusing on sesquiterpene lactones.
**Figure S5:** Transcriptome profiling of chicory root tissues.
**Figure S6:** Overview of transcriptomics data.
**Figure S7:** Expression data of selected pathways and gene families.
**Figure S8:** Unrooted phylogenetic tree of *cis*‐prenyltransferases including chicory CPT candidates and CPT genes that were described to be involved in rubber biosynthesis.
**Figure S9:** Unrooted phylogenetic tree of chicory cytochrome P450 oxygenases (Ci‐CYP) candidates that are overexpressed in the latex.
**Figure S10:** Verification of the tissues specific expression of a sample chicory genes by RT‐qPCR.
**Table S1:** Parameters and peak area for targeted STL measurements.
**Table S6:** Inulin and sugar analysis.

**Table S2:** Peak areas of untargeted profiling MS data.

**Table S3:** RNAseq expression data.

**Table S4:** KEGG pathway enrichment for RNASeq and metabolomics data.

**Table S5:** Selected list of differentially expressed genes.

## Data Availability

The transcriptome data presented in this study are deposited in the NCBI repository under accession numbers PRJNA824299. The metabolome data are deposited in the Metabolights database under the accession number MTBLS6917 (www.ebi.ac.uk/metabolights/MTBLS6917).

## References

[ppl70778-bib-0001] Balcke, G. U. , S. Bennewitz , N. Bergau , et al. 2017. “Multi‐Omics of Tomato Glandular Trichomes Reveals Distinct Features of Central Carbon Metabolism Supporting High Productivity of Specialized Metabolites.” Plant Cell 29: 960–983. 28408661 10.1105/tpc.17.00060 PMC5466034

[ppl70778-bib-0002] Balcke, G. U. , V. Handrick , N. Bergau , et al. 2012. “An UPLC‐MS/MS Method for Highly Sensitive High‐Throughput Analysis of Phytohormones in Plant Tissues.” Plant Methods 8: 47. 23173950 10.1186/1746-4811-8-47 PMC3573895

[ppl70778-bib-0003] Banasiak, J. , T. Jamruszka , J. D. Murray , and M. Jasiński . 2021. “A Roadmap of Plant Membrane Transporters in Arbuscular Mycorrhizal and Legume–Rhizobium Symbioses.” Plant Physiology 187: 2071–2091. 34618047 10.1093/plphys/kiab280 PMC8644718

[ppl70778-bib-0004] Benninghaus, V. A. , N. van Deenen , B. Müller , et al. 2020. “Comparative Proteome and Metabolome Analyses of Latex‐Exuding and Non‐Exuding Taraxacum Koksaghyz Roots Provide Insights Into Laticifer Biology.” Journal of Experimental Botany 71: 1278–1293. 31740929 10.1093/jxb/erz512 PMC7031084

[ppl70778-bib-0005] Bogdanović, M. , K. Cankar , S. Todorović , et al. 2019. “Tissue Specific Expression and Genomic Organization of Bitter Sesquiterpene Lactone Biosynthesis in *Cichorium Intybus* L. ‐ Asteraceae.” Industrial Crops and Products 129: 253–260.

[ppl70778-bib-0006] Bolger, A. M. , M. Lohse , and B. Usadel . 2014. “Trimmomatic: A Flexible Trimmer for Illumina Sequence Data.” Bioinformatics 30: 2114–2120. 24695404 10.1093/bioinformatics/btu170 PMC4103590

[ppl70778-bib-0007] Bouwmeester, H. J. , J. Kodde , F. W. A. Verstappen , I. G. Altug , J.‐W. de Kraker , and T. E. Wallaart . 2002. “Isolation and Characterization of Two Germacrene A Synthase cDNA Clones From Chicory.” Plant Physiology 129: 134–144. 12011345 10.1104/pp.001024 PMC155878

[ppl70778-bib-0008] Cahill, G. F., Jr. 2006. “Fuel Metabolism in Starvation.” Annual Review of Nutrition 26: 1–22. 10.1146/annurev.nutr.26.061505.111258 16848698

[ppl70778-bib-0009] Cankar, K. , P. Bundock , R. Sevenier , et al. 2021. “Inactivation of the Germacrene A Synthase Genes by CRISPR/Cas9 Eliminates the Biosynthesis of Sesquiterpene Lactones in *Cichorium Intybus* L.” Plant Biotechnology Journal 19: 2442–2453. 34270859 10.1111/pbi.13670 PMC8633505

[ppl70778-bib-0010] Cankar, K. , J. C. Hakkert , R. Sevenier , et al. 2022. “CRISPR ‐ Cas9 Targeted Inactivation of the Kauniolide Synthase in Chicory Results in Accumulation of Costunolide and Its Conjugates in Taproots.” Frontiers in Plant Science 13: 940003. 36105709 10.3389/fpls.2022.940003 PMC9465254

[ppl70778-bib-0011] Cankar, K. , J. C. Hakkert , R. Sevenier , et al. 2023. “Lactucin Synthase Inactivation Boosts the Accumulation of Anti‐Inflammatory 8‐Deoxylactucin and Its Derivatives in Chicory ‐ *Cichorium intybus* L.” Journal of Agricultural and Food Chemistry 71: 6061–6072. 37036799 10.1021/acs.jafc.2c08959 PMC10119987

[ppl70778-bib-0012] Cao, M. , D. Wang , Y. Mao , et al. 2017. “Integrating Transcriptomics and Metabolomics to Characterize the Regulation of EPA Biosynthesis in Response to Cold Stress in Seaweed Bangia Fuscopurpurea.” PLoS One 12: e0186986. 29240755 10.1371/journal.pone.0186986 PMC5730106

[ppl70778-bib-0013] Cao, Y. , J. Zhai , Q. Wang , H. Yuan , and X. Huang . 2017. “Function of *Hevea brasiliensis* NAC1 in Dehydration‐Induced Laticifer Differentiation and Latex Biosynthesis.” Planta 245: 31–44. 27544199 10.1007/s00425-016-2589-0

[ppl70778-bib-0014] Chen, X. , J. M. Hagel , L. Chang , et al. 2018. “A Pathogenesis‐Related 10 Protein Catalyzes the Final Step in Thebaine Biosynthesis.” Nature Chemical Biology 14: 738–743. 29807982 10.1038/s41589-018-0059-7

[ppl70778-bib-0015] Chen, Y.‐Y. , L.‐F. Wang , L.‐J. Dai , S.‐G. Yang , and W.‐M. Tian . 2011. “Characterization of HbEREBP1, A Wound‐Responsive Transcription Factor Gene in Laticifers of *Hevea brasiliensis* Muell. Arg.” Molecular Biology Reports 39: 3713–3719. 21761140 10.1007/s11033-011-1146-y

[ppl70778-bib-0016] Cherian, S. , S. B. Ryu , and K. Cornish . 2019. “Natural Rubber Biosynthesis in Plants, the Rubber Transferase Complex, and Metabolic Engineering Progress and Prospects.” Plant Biotechnology Journal 17: 2041–2061. 31150158 10.1111/pbi.13181 PMC6790360

[ppl70778-bib-0017] Conesa, A. , S. Gotz , J. M. Garcia‐Gomez , J. Terol , M. Talon , and M. Robles . 2005. “Blast2GO: A Universal Tool for Annotation, Visualization and Analysis in Functional Genomics Research.” Bioinformatics 21: 3674–3676. 16081474 10.1093/bioinformatics/bti610

[ppl70778-bib-0018] Conesa, A. , P. Madrigal , S. Tarazona , et al. 2016. “A Survey of Best Practices for RNA‐Seq Data Analysis.” Genome Biology 17: 13. 26813401 10.1186/s13059-016-0881-8 PMC4728800

[ppl70778-bib-0019] Darwen, C. W. , and P. John . 1989. “Localization of the Enzymes of Fructan Metabolism in Vacuoles Isolated by a Mechanical Method From Tubers of Jerusalem Artichoke ‐ *Helianthus tuberosus* L.” Plant Physiology 89: 658–663. 16666597 10.1104/pp.89.2.658 PMC1055897

[ppl70778-bib-0020] Dastmalchi, M. , L. Chang , R. Chen , et al. 2019. “Purine Permease‐Type Benzylisoquinoline Alkaloid Transporters in Opium Poppy.” Plant Physiology 181: 916–933. 31467164 10.1104/pp.19.00565 PMC6836811

[ppl70778-bib-8001] de Kraker, J.‐W. , M. C. R. Franssen , A. de Groot , W. A. König , and H. J. Bouwmeester . 1998. “(+)‐Germacrene A Biosynthesis.” Plant Physiology 117, no. 4: 1381–1392. 10.1104/pp.117.4.1381. 9701594 PMC34902

[ppl70778-bib-0021] Djoumbou Feunang, Y. , R. Eisner , C. Knox , et al. 2016. “ClassyFire: Automated Chemical Classification With a Comprehensive, Computable Taxonomy.” Journal of Cheminformatics 8: 61. 27867422 10.1186/s13321-016-0174-y PMC5096306

[ppl70778-bib-0022] Dobin, A. , C. A. Davis , F. Schlesinger , et al. 2013. “STAR: Ultrafast Universal RNA‐Seq Aligner.” Bioinformatics 29: 15–21. 23104886 10.1093/bioinformatics/bts635 PMC3530905

[ppl70778-bib-0023] Duan, C. , M. Rio , J. Leclercq , F. Bonnot , G. Oliver , and P. Montoro . 2010. “Gene Expression Pattern in Response to Wounding, Methyl Jasmonate and Ethylene in the Bark of *Hevea brasiliensis* .” Tree Physiology 30: 1349–1359. 20660491 10.1093/treephys/tpq066

[ppl70778-bib-0024] Epping, J. , N. van Deenen , E. Niephaus , et al. 2015. “A Rubber Transferase Activator Is Necessary for Natural Rubber Biosynthesis in Dandelion.” Nature Plants 1: 15048.

[ppl70778-bib-0025] Frey, M. , I. Klaiber , J. Conrad , et al. 2019. “Characterization of CYP71AX36 From Sunflower ‐ *Helianthus Annuus* L., Asteraceae.” Scientific Reports 9: 14295. 31586110 10.1038/s41598-019-50520-6 PMC6778120

[ppl70778-bib-0026] Frey, M. , I. Klaiber , J. Conrad , and O. Spring . 2020. “CYP71BL9, The Missing Link in Costunolide Synthesis of Sunflower.” Phytochemistry 177: 112430. 32516579 10.1016/j.phytochem.2020.112430

[ppl70778-bib-0027] Frey, M. , K. Schmauder , I. Pateraki , and O. Spring . 2018. “Biosynthesis of Eupatolide‐A Metabolic Route for Sesquiterpene Lactone Formation Involving the P450 Enzyme CYP71DD6.” ACS Chemical Biology 13: 1536–1543. 29758164 10.1021/acschembio.8b00126

[ppl70778-bib-0028] Goldstone, J. V. 2008. “Environmental Sensing and Response Genes in Cnidaria: The Chemical Defensome in the Sea Anemone *Nematostella vectensis* .” Cell Biology and Toxicology 24: 483–502. 18956243 10.1007/s10565-008-9107-5 PMC2811067

[ppl70778-bib-0029] Grabherr, M. G. , B. J. Haas , M. Yassour , et al. 2011. “Full‐Length Transcriptome Assembly From RNA‐Seq Data Without a Reference Genome.” Nature Biotechnology 29: 644–652. 10.1038/nbt.1883 PMC3571712 21572440

[ppl70778-bib-0030] Häkkinen, S. T. , M. Soković , L. Nohynek , et al. 2021. “Chicory Extracts and Sesquiterpene Lactones Show Potent Activity Against Bacterial and Fungal Pathogens.” Pharmaceuticals ‐ Basel 14: 941. 34577641 10.3390/ph14090941 PMC8469098

[ppl70778-bib-0031] Hao, B. 2000. “Laticifer Differentiation in *Hevea brasiliensis*: Induction by Exogenous Jasmonic Acid and Linolenic Acid.” Annals of Botany 85: 37–43.

[ppl70778-bib-0032] Huang, D. W. , B. T. Sherman , and R. A. Lempicki . 2008. “Bioinformatics Enrichment Tools: Paths Toward the Comprehensive Functional Analysis of Large Gene Lists.” Nucleic Acids Research 37: 1–13. 19033363 10.1093/nar/gkn923 PMC2615629

[ppl70778-bib-0033] Huber, M. , Z. Bont , J. Fricke , et al. 2016. “A Below‐Ground Herbivore Shapes Root Defensive Chemistry in Natural Plant Populations.” Proceedings of the Royal Society B: Biological Sciences 283: 20160285. 10.1098/rspb.2016.0285 PMC4822473 27009228

[ppl70778-bib-0034] Huber, M. , J. Epping , C. Schulze Gronover , et al. 2016. “A Latex Metabolite Benefits Plant Fitness under Root Herbivore Attack.” PLoS Biology 14: e1002332. 26731567 10.1371/journal.pbio.1002332 PMC4701418

[ppl70778-bib-8002] Ikezawa, N. , D. T. Nguyen , S.‐U. Kim , P. E. O’Maille , O. Spring , and D.‐K. Ro . 2011. “Lettuce Costunolide Synthase (CYP71BL2) and Its Homolog (CYP71BL1) from Sunflower Catalyze Distinct Regio‐ and Stereoselective Hydroxylations in Sesquiterpene Lactone Metabolism.” Journal of Biological Chemistry 286, no. 24: 21601–21611. 10.1074/jbc.m110.216804. 21515683 PMC3122218

[ppl70778-bib-0035] Jasinski, M. , Y. Stukkens , H. Degand , B. Purnelle , J. Marchand‐Brynaert , and M. Boutry . 2001. “A Plant Plasma Membrane ATP Binding Cassette‐Type Transporter Is Involved in Antifungal Terpenoid Secretion.” Plant Cell 13: 1095–1108. 11340184 PMC135550

[ppl70778-bib-0036] Kanehisa, M. 2000. “KEGG: Kyoto Encyclopedia of Genes and Genomes.” Nucleic Acids Research 28: 27–30. 10592173 10.1093/nar/28.1.27 PMC102409

[ppl70778-bib-0037] Kanehisa, M. , and Y. Sato . 2019. “KEGG Mapper for Inferring Cellular Functions From Protein Sequences.” Protein Science 29: 28–35. 31423653 10.1002/pro.3711 PMC6933857

[ppl70778-bib-0038] Kitajima, S. , W. Aoki , D. Shibata , et al. 2018. “Comparative Multi‐Omics Analysis Reveals Diverse Latex‐Based Defense Strategies Against Pests Among Latex‐Producing Organs of the Fig Tree ‐ Ficus carica .” Planta 247: 1423–1438. 29536219 10.1007/s00425-018-2880-3

[ppl70778-bib-0039] Koonin, E. V. , N. D. Fedorova , J. D. Jackson , et al. 2004. “A Comprehensive Evolutionary Classification of Proteins Encoded in Complete Eukaryotic Genomes.” Genome Biology 5: R7. 14759257 10.1186/gb-2004-5-2-r7 PMC395751

[ppl70778-bib-0040] Kwon, M. , C. L. Hodgins , T. M. Haslam , et al. 2022. “Germacrene A Synthases for Sesquiterpene Lactone Biosynthesis Are Expressed in Vascular Parenchyma Cells Neighboring Laticifers in Lettuce.” Plants 11: 1192. 35567193 10.3390/plants11091192 PMC9099558

[ppl70778-bib-0041] Langmead, B. , and S. L. Salzberg . 2012. “Fast Gapped‐Read Alignment With Bowtie 2.” Nature Methods 9: 357–359. 22388286 10.1038/nmeth.1923 PMC3322381

[ppl70778-bib-0042] Laosombut, T. , P. Arreewichit , K. Nirapathpongporn , et al. 2016. “Differential Expression of Methyl Jasmonate‐Responsive Genes Correlates With Laticifer Vessel Proliferation in Phloem Tissue of Rubber Tree—*Hevea brasiliensis* .” Journal of Plant Growth Regulation 35: 1049–1063.

[ppl70778-bib-0043] Li, B. , and C. N. Dewey . 2011. “RSEM: Accurate Transcript Quantification From RNA‐Seq Data With or Without a Reference Genome.” BMC Bioinformatics 12: 323. 21816040 10.1186/1471-2105-12-323 PMC3163565

[ppl70778-bib-0044] Li, R. , H. Shang , H. Wu , M. Wang , M. Duan , and J. Yang . 2018. “Thermal Inactivation Kinetics and Effects of Drying Methods on the Phenolic Profile and Antioxidant Activities of Chicory—*Cichorium Intybus* L. Leaves.” Scientific Reports 8: 9529. 29934537 10.1038/s41598-018-27874-4 PMC6015010

[ppl70778-bib-0045] Liu, Q. , A. Beyraghdar Kashkooli , D. Manzano , et al. 2018. “Kauniolide Synthase Is a P450 With Unusual Hydroxylation and Cyclization‐Elimination Activity.” Nature Communications 9: 4657. 10.1038/s41467-018-06565-8 PMC6220293 30405138

[ppl70778-bib-0046] Liu, Q. , D. Manzano , N. Tanić , et al. 2014. “Elucidation and in Planta Reconstitution of the Parthenolide Biosynthetic Pathway.” Metabolic Engineering 23: 145–153. 24704560 10.1016/j.ymben.2014.03.005

[ppl70778-bib-0047] Loh, S. C. , A. S. Othman , and G. Veera Singham . 2019. “Identification and Characterization of Jasmonic Acid‐ and Linolenic Acid‐Mediated Transcriptional Regulation of Secondary Laticifer Differentiation in *Hevea brasiliensis* .” Scientific Reports 9: 14296. 31586098 10.1038/s41598-019-50800-1 PMC6778104

[ppl70778-bib-0048] Maeda, Y. , and T. Kinoshita . 2011. “Structural Remodeling, Trafficking and Functions of Glycosylphosphatidylinositol‐Anchored Proteins.” Progress in Lipid Research 50: 411–424. 21658410 10.1016/j.plipres.2011.05.002

[ppl70778-bib-0049] Makita, Y. , K. K. Ng , G. Veera Singham , et al. 2017. “Large‐Scale Collection of Full‐Length cDNA and Transcriptome Analysis In *Hevea brasiliensis* .” DNA Research 24, no. 2: 159–167. 28431015 10.1093/dnares/dsw056 PMC5397604

[ppl70778-bib-0050] Maroufi, A. , E. Van Bockstaele , and M. De Loose . 2010. “Validation of Reference Genes for Gene Expression Analysis in Chicory—*Cichorium Intybus* Using Quantitative Real‐Time PCR.” BMC Molecular Biology 11: 15. 20156357 10.1186/1471-2199-11-15 PMC2830926

[ppl70778-bib-0051] Matos, M. S. , J. D. Anastácio , J. W. Allwood , et al. 2020. “Assessing the Intestinal Permeability and Anti‐Inflammatory Potential of Sesquiterpene Lactones From Chicory.” Nutrients 12: 3547. 33228214 10.3390/nu12113547 PMC7699524

[ppl70778-bib-0052] Natarajan, P. , T. A. Akinmoju , P. Nimmakayala , et al. 2020. “Integrated Metabolomic and Transcriptomic Analysis to Characterize Cutin Biosynthesis Between Low‐ and High‐Cutin Genotypes of *Capsicum Chinense* Jacq.” International Journal of Molecular Sciences 21: 1397. 32092953 10.3390/ijms21041397 PMC7073079

[ppl70778-bib-0053] Newman, J. C. , and E. Verdin . 2014. “Ketone Bodies as Signaling Metabolites.” Trends in Endocrinology and Metabolism 25: 42–52. 24140022 10.1016/j.tem.2013.09.002 PMC4176946

[ppl70778-bib-0054] Nguyen, D. T. , J. C. Göpfert , N. Ikezawa , et al. 2010. “Biochemical Conservation and Evolution of Germacrene A Oxidase in Asteraceae*.” Journal of Biological Chemistry 285: 16588–16598. 20351109 10.1074/jbc.M110.111757 PMC2878029

[ppl70778-bib-0055] Onoyovwe, A. , J. M. Hagel , X. Chen , M. F. Khan , D. C. Schriemer , and P. J. Facchini . 2013. “Morphine Biosynthesis in Opium Poppy Involves Two Cell Types: Sieve Elements and Laticifers.” Plant Cell 25: 4110–4122. 24104569 10.1105/tpc.113.115113 PMC3877807

[ppl70778-bib-0056] Ozber, N. , and P. J. Facchini . 2022. “Phloem‐Specific Localization of Benzylisoquinoline Alkaloid Metabolism in Opium Poppy.” Journal of Plant Physiology 271: 153641. 35240512 10.1016/j.jplph.2022.153641

[ppl70778-bib-0057] Paterlini, A. 2020. “Uncharted Routes: Exploring the Relevance of Auxin Movement via Plasmodesmata.” Biology Open 9, no. 11: bio055541. 33184092 10.1242/bio.055541 PMC7673358

[ppl70778-bib-0058] Peña‐Espinoza, M. , U. Boas , A. R. Williams , S. M. Thamsborg , H. T. Simonsen , and H. L. Enemark . 2015. “Sesquiterpene Lactone Containing Extracts From Two Cultivars of Forage Chicory— *Cichorium intybus* Show Distinctive Chemical Profiles and in Vitro Activity Against Ostertagia Ostertagi.” International Journal for Parasitology: Drugs and Drug Resistance 5: 191–200. 27120066 10.1016/j.ijpddr.2015.10.002 PMC4847107

[ppl70778-bib-0059] Peña‐Espinoza, M. , A. H. Valente , L. Bornancin , et al. 2020. “Anthelmintic and Metabolomic Analyses of Chicory (*Cichorium Intybus*) Identify an Industrial By‐Product With Potent In Vitro Antinematodal Activity.” Veterinary Parasitology 280: 109088. 32278938 10.1016/j.vetpar.2020.109088

[ppl70778-bib-0060] Philippe, G. , D. De Bellis , J. K. C. Rose , and C. Nawrath . 2022. “Trafficking Processes and Secretion Pathways Underlying the Formation of Plant Cuticles.” Frontiers in Plant Science 12: 786874. 35069645 10.3389/fpls.2021.786874 PMC8769167

[ppl70778-bib-0061] Pütter, K. M. , N. van Deenen , K. Unland , D. Prüfer , and C. Schulze Gronover . 2017. “Isoprenoid Biosynthesis in Dandelion Latex Is Enhanced by the Overexpression of Three Key Enzymes Involved in the Mevalonate Pathway.” BMC Plant Biology 17: 88. 28532507 10.1186/s12870-017-1036-0 PMC5441070

[ppl70778-bib-0062] Qu, Y. , R. Chakrabarty , H. T. Tran , et al. 2015. “A Lettuce ‐ *Lactuca Sativa* Homolog of Human Nogo‐B Receptor Interacts With Cis‐Prenyltransferase and Is Necessary for Natural Rubber Biosynthesis*.” Journal of Biological Chemistry 290: 1898–1914. 25477521 10.1074/jbc.M114.616920 PMC4303647

[ppl70778-bib-0063] Quigg, A. , and J. Beardall . 2003. “Protein Turnover in Relation to Maintenance Metabolism at Low Photon Flux in Two Marine Microalgae.” Plant, Cell & Environment 26: 693–703.

[ppl70778-bib-0064] Robinson, M. D. , D. J. McCarthy , and G. K. Smyth . 2009. “Edger: A Bioconductor Package for Differential Expression Analysis of Digital Gene Expression Data.” Bioinformatics 26: 139–140. 19910308 10.1093/bioinformatics/btp616 PMC2796818

[ppl70778-bib-0065] Schulze, C. , D. Wahler , and D. Prufer . 2011. “Natural Rubber Biosynthesis and Physic‐Chemical Studies on Plant Derived Latex.” In Biotechnology of Biopolymers. InTech.

[ppl70778-bib-0066] Seo, M. W. , D. S. Yang , S. J. Kays , G. P. Lee , and K. W. Park . 2009. “Sesquiterpene Lactones and Bitterness in Korean Leaf Lettuce Cultivars.” HortScience 44: 246–249.

[ppl70778-bib-0067] Sessa, R. A. , M. H. Bennett , M. J. Lewis , J. W. Mansfield , and M. H. Beale . 2000. “Metabolite Profiling of Sesquiterpene Lactones From Lactuca Species ‐ Major Latex Components Are Novel Oxalate and Sulfate Conjugates of Lactucin and Its Derivatives.” Journal of Biological Chemistry 275: 26877–26884. 10858433 10.1074/jbc.M000244200

[ppl70778-bib-0068] Sharma, J. , L. Balakrishnan , S. Kaushik , and M. K. Kashyap . 2020. “Editorial: Multi‐Omics Approaches to Study Signaling Pathways.” Frontiers in Bioengineering and Biotechnology 8: 829. 33014991 10.3389/fbioe.2020.00829 PMC7499333

[ppl70778-bib-0069] Sheard, L. B. , X. Tan , H. Mao , et al. 2010. “Jasmonate Perception by Inositol‐Phosphate‐Potentiated COI1–JAZ Co‐Receptor.” Nature 468: 400–405. 20927106 10.1038/nature09430 PMC2988090

[ppl70778-bib-0070] Shyu, C. , P. Figueroa , C. L. DePew , et al. 2012. “JAZ8 Lacks a Canonical Degron and Has an EAR Motif That Mediates Transcriptional Repression of Jasmonate Responses in *Arabidopsis* .” Plant Cell 24: 536–550. 22327740 10.1105/tpc.111.093005 PMC3315231

[ppl70778-bib-0071] Stoop, J. M. , J. Van Arkel , J. C. Hakkert , C. Tyree , P. G. Caimi , and A. J. Koops . 2007. “Developmental Modulation of Inulin Accumulation in Storage Organs of Transgenic Maize and Transgenic Potato.” Plant Science 173: 172–181.

[ppl70778-bib-0072] Taj, G. , P. Agarwal , M. Grant , and A. Kumar . 2010. “MAPK Machinery in Plants: Recognition and Response to Different Stresses Through Multiple Signal Transduction Pathways.” Plant Signaling & Behavior 5: 1370–1378. 20980831 10.4161/psb.5.11.13020 PMC3115236

[ppl70778-bib-0073] Tan, D. , X. Sun , and J. Zhang . 2014. “Age‐Dependent and Jasmonic Acid‐Induced Laticifer‐Cell Differentiation in Anther Callus Cultures of Rubber Tree.” Planta 240: 337–344. 24841475 10.1007/s00425-014-2086-2

[ppl70778-bib-0074] Tang, C. , X. Xiao , H. Li , et al. 2013. “Comparative Analysis of Latex Transcriptome Reveals Putative Molecular Mechanisms Underlying Super Productivity of *Hevea Brasiliensis* .” PLoS One 8: e75307‐e75307. 24066172 10.1371/journal.pone.0075307 PMC3774812

[ppl70778-bib-0075] Teoh, K. H. , D. R. Polichuk , D. W. Reed , G. Nowak , and P. S. Covello . 2006. “ *Artemisia annua* L. ‐ Asteraceae Trichome‐Specific cDNAs Reveal CYP71AV1, a Cytochrome P450 With a Key Role in the Biosynthesis of the Antimalarial Sesquiterpene Lactone Artemisinin.” FEBS Letters 580: 1411–1416. 16458889 10.1016/j.febslet.2006.01.065

[ppl70778-bib-0076] Testone, G. , G. Mele , E. Di Giacomo , et al. 2017. “Corrigendum: Insights Into the Sesquiterpenoid Pathway by Metabolic Profiling and De Novo Transcriptome Assembly of Stem‐Chicory ‐ *Cichorium Intybus* Cultigroup “Catalogna”.” Frontiers in Plant Science 8: 00478. 10.3389/fpls.2017.00478 PMC5383726 28396680

[ppl70778-bib-0077] Testone, G. , A. Sobolev , M. Gonnella , et al. 2019. “Insights Into Sucrose Pathway of Chicory Stems by Integrative Transcriptomic and Metabolic Analyses.” Phytochemistry 167: 112086. 31450092 10.1016/j.phytochem.2019.112086

[ppl70778-bib-0078] Tian, W.‐M. , S.‐G. Yang , M.‐J. Shi , S.‐X. Zhang , and J.‐L. Wu . 2015. “Mechanical Wounding‐Induced Laticifer Differentiation in Rubber Tree: An Indicative Role of Dehydration, Hydrogen Peroxide, and Jasmonates.” Journal of Plant Physiology 182: 95–103. 26070085 10.1016/j.jplph.2015.04.010

[ppl70778-bib-0079] Treutler, H. , H. Tsugawa , A. Porzel , et al. 2016. “Discovering Regulated Metabolite Families in Untargeted Metabolomics Studies.” Analytical Chemistry 88: 8082–8090. 27452369 10.1021/acs.analchem.6b01569

[ppl70778-bib-0080] Tsugawa, H. , T. Cajka , T. Kind , et al. 2015. “MS‐DIAL: Data‐Independent MS/MS Deconvolution for Comprehensive Metabolome Analysis.” Nature Methods 12: 523–526. 25938372 10.1038/nmeth.3393 PMC4449330

[ppl70778-bib-0081] Tsujikawa, Y. , S. Ishikawa , I. Sakane , K.‐i. Yoshida , and R. Osawa . 2021. “Identification of Genes Encoding a Novel ABC Transporter in *Lactobacillus Delbrueckii* for Inulin Polymers Uptake.” Scientific Reports 11: 16007. 34362962 10.1038/s41598-021-95356-1 PMC8346543

[ppl70778-bib-0082] Tungngoen, K. , P. Kongsawadworakul , U. Viboonjun , et al. 2009. “Involvement of HbPIP2;1 and HbTIP1;1 Aquaporins in Ethylene Stimulation of Latex Yield Through Regulation of Water Exchanges Between Inner Liber and Latex Cells in *Hevea brasiliensis* .” Plant Physiology 151: 843–856. 19656906 10.1104/pp.109.140228 PMC2754619

[ppl70778-bib-0083] Verhoeven, K. J. F. , E. H. Verbon , T. P. van Gurp , et al. 2017. “Intergenerational Environmental Effects: Functional Signals in Offspring Transcriptomes and Metabolomes After Parental Jasmonic Acid Treatment in Apomictic Dandelion.” New Phytologist 217: 871–882. 29034954 10.1111/nph.14835 PMC5741498

[ppl70778-bib-0084] Vierstra, R. D. 1993. “Protein Degradation in Plants.” Annual Review of Plant Physiology and Plant Molecular Biology 44: 385–410.

[ppl70778-bib-0085] Wahler, D. , C. S. Gronover , C. Richter , et al. 2009. “Polyphenoloxidase Silencing Affects Latex Coagulation in Taraxacum Species.” Plant Physiology 151: 334–346. 19605551 10.1104/pp.109.138743 PMC2736003

[ppl70778-bib-0086] Woolsey, I. D. , A. H. Valente , A. R. Williams , S. M. Thamsborg , H. T. Simonsen , and H. L. Enemark . 2019. “Anti‐Protozoal Activity of Extracts From Chicory ‐ *Cichorium Intybus* ‐ Against *Cryptosporidium Parvum* in Cell Culture.” Scientific Reports 9: 20414. 31892721 10.1038/s41598-019-56619-0 PMC6938481

[ppl70778-bib-0087] Wu, L. , Y. Guan , Z. Wu , et al. 2014. “OsABCG15 Encodes a Membrane Protein That Plays an Important Role in Anther Cuticle and Pollen Exine Formation in Rice.” Plant Cell Reports 33: 1881–1899. 25138437 10.1007/s00299-014-1666-8 PMC4197380

[ppl70778-bib-0088] Xiao, L. , H. X. Tan , and L. Zhang . 2016. “ *Artemisia Annua* Glandular Secretory Trichomes: The Biofactory of Antimalarial Agent Artemisinin.” Science Bulletin 61: 26–36.

[ppl70778-bib-0089] Xie, Q. , L. Ma , P. Tan , et al. 2020. “Multiple High‐Affinity K+ Transporters and ABC Transporters Involved in K+ Uptake/Transport in the Potassium‐Hyperaccumulator Plant *Phytolacca Acinosa* Roxb.” Plants 9: 470. 32276334 10.3390/plants9040470 PMC7238005

[ppl70778-bib-0090] Xin, A. , and K. Herburger . 2021. “Mini Review: Transport of Hydrophobic Polymers Into the Plant Apoplast.” Frontiers in Plant Science 11: 590990. 33488642 10.3389/fpls.2020.590990 PMC7817615

[ppl70778-bib-0091] Yang, Z.‐P. , H.‐L. Li , D. Guo , W.‐M. Tian , and S.‐Q. Peng . 2011. “Molecular Characterization of a Novel 14‐3‐3 Protein Gene (Hb14‐3‐3c) From *Hevea brasiliensis* .” Molecular Biology Reports 39: 4491–4497. 21947841 10.1007/s11033-011-1239-7

[ppl70778-bib-0092] Zhang, Q. , D. Wang , H. Zhang , et al. 2018. “Detection of Autophagy Processes During the Development of Nonarticulated Laticifers in *Euphorbia kansui* Liou.” Planta 247: 845–861. 29260395 10.1007/s00425-017-2835-0

[ppl70778-bib-0093] Zhang, S. , S. Wu , and W. Tian . 2016. “The Secondary Laticifer Differentiation in Rubber Tree Is Induced by Trichostatin A, An Inhibitor of Histone Acetylation.” Frontiers of Agricultural Science and Engineering 3: 357.

[ppl70778-bib-0094] Zhao, Y. , L.‐M. Zhou , Y.‐Y. Chen , S.‐G. Yang , and W.‐M. Tian . 2011. “MYC Genes With Differential Responses to Tapping, Mechanical Wounding, Ethrel and Methyl Jasmonate in Laticifers of Rubber Tree (*Hevea brasiliensis* Muell. Arg.).” Journal of Plant Physiology 168: 1649–1658. 21489651 10.1016/j.jplph.2011.02.010

