# Supplementary material for: Metabolome and Transcriptome Profiling of Chicory Roots Provide Insights Into Laticifer Development and Specialized Metabolism
Source: Physiol Plant. 2026 Feb 8;178(1):e70778. doi: 10.1111/ppl.70778 (PMC12884033; doi:10.1111/ppl.70778)
Supplement: Supplementary file 1 — Figure S1: Chicory root tissue visualization. Figure S2: Principal component analysis of STLs in chicory roots. Figure S3: Overview of the metabolomics data from chicory roots. Figure S4: Excerpt of a MetFamily output focusing on sesquiterpene lactones. Figure S5: Transcriptome profiling of chicory root tissues. Figure S6: Overview of transcriptomics data. Figure S7: Expression data of selected pathways and gene families. Figure S8: Unrooted phylogenetic tree of cis‐prenyltransferases including chicory CPT candidates and CPT genes that were described to be involved in rubber biosynthesis. Figure S9: Unrooted phylogenetic tree of chicory cytochrome P450 oxygenases (Ci‐CYP) candidates that are overexpressed in the latex. Figure S10: Verification of the tissues specific expression of a sample chicory genes by RT‐qPCR. Table S1: Parameters and peak area for targeted STL measurements. Table S6: Inulin and sugar analysis. [file PPL-178-e70778-s001.pdf]

Metabolome and transcriptome profiling of chicory roots provide insights into laticifer development and specialized metabolism

Khabat Vahabi, Gerd U. Balcke, Johanna C. Hakkert, Ingrid M. van der Meer, Benedikt Athmer and Alain Tissier

Supplementary Figures S1-S10

Supplementary Tables S1 and S6

Supplementary Tables S2 to S5 are available as separate files due to their large size

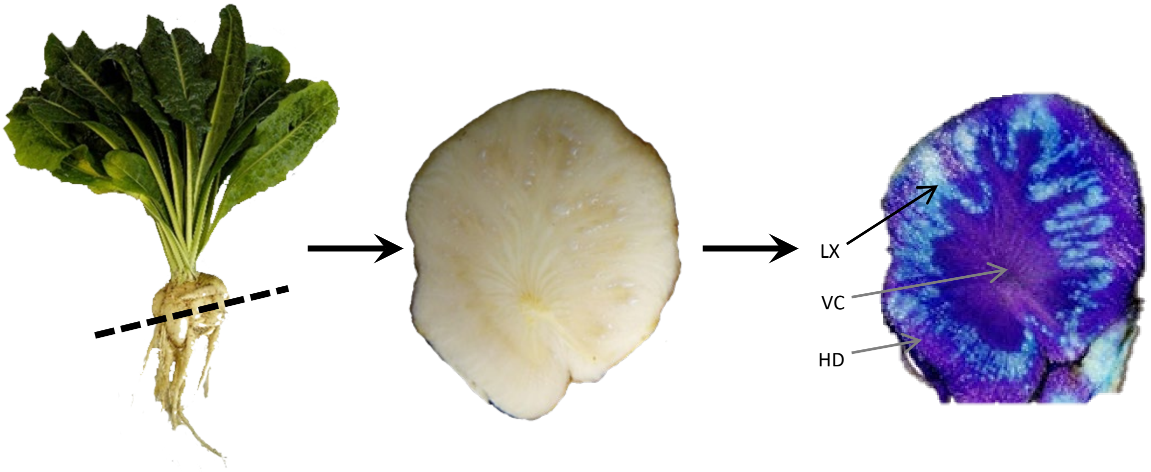

Figure S1. Chicory root tissue visualization. TBO staining (image on the right side) of the horizontal section of the chicory root and demonstration of the populated area with different tissues of the taproot including latex (LX), hypodermis (HD) and vascular cylinder (VC).

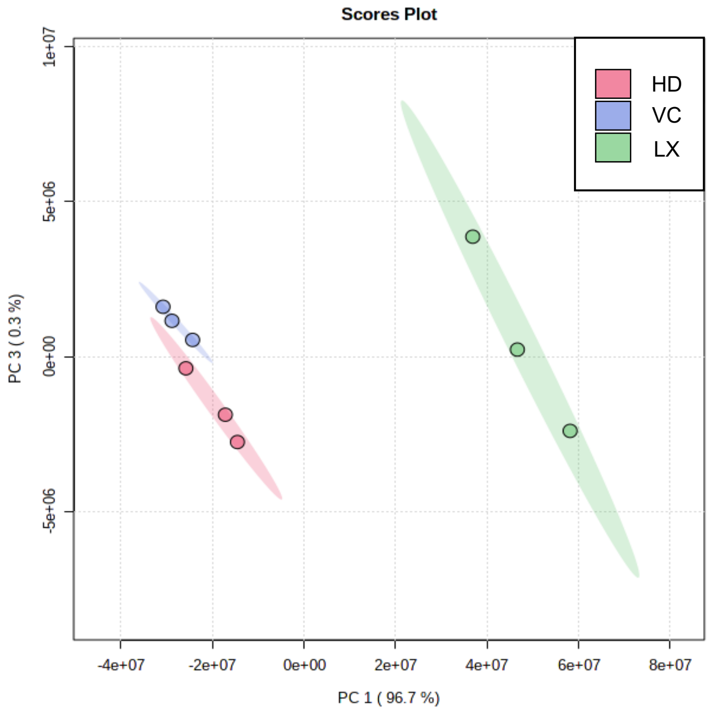

Figure S2. Principal component analysis of STLs in chicory roots. Scores plot of principal component analysis of biological repeats across different cell types based on the peak area of the major STLs using LC-MS positive mode (LC-MS-Pos). HD: hypodermis; VC: vascular cylinder; LX: latex.

A

	Annotated metabolite in cell types			annotation		
	HD	LX	VC	total detected	annotated	unknown
GC-MS	39	289	83	1657	330	1327
LC-MS-Neg	53	78	55	3195	99	3096
LC-MS-Pos	85	92	84	3631	112	3519
LC-MS-hpm	200	298	246	12954	337	12617
total	377	757	468	21437	878	20559

B

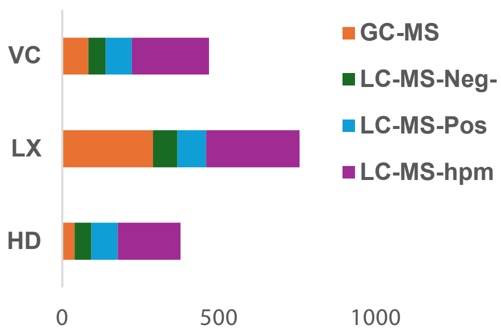

C

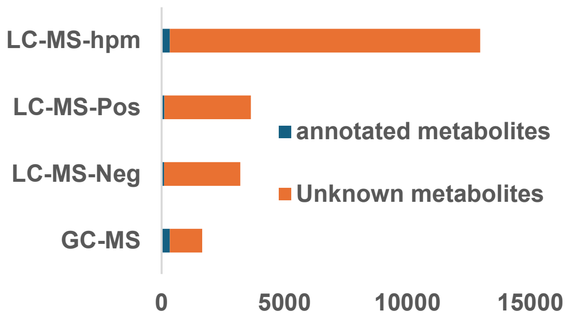

Figure S3. Overview of the metabolomics data from chicory roots. A. Table summarizing the number of features detected in different chicory root tissues by various MS methods and the number of annotated and unknown compounds. HD: hypodermis; LX: latex; VC: vascular cylinder. **B**. Graphical representation of the metabolites measured in different cell types using various MS methods. **C.** Graphical representation of the annotated and unknown metabolites across various MS methods. hydrophilic metabolites (hpm)

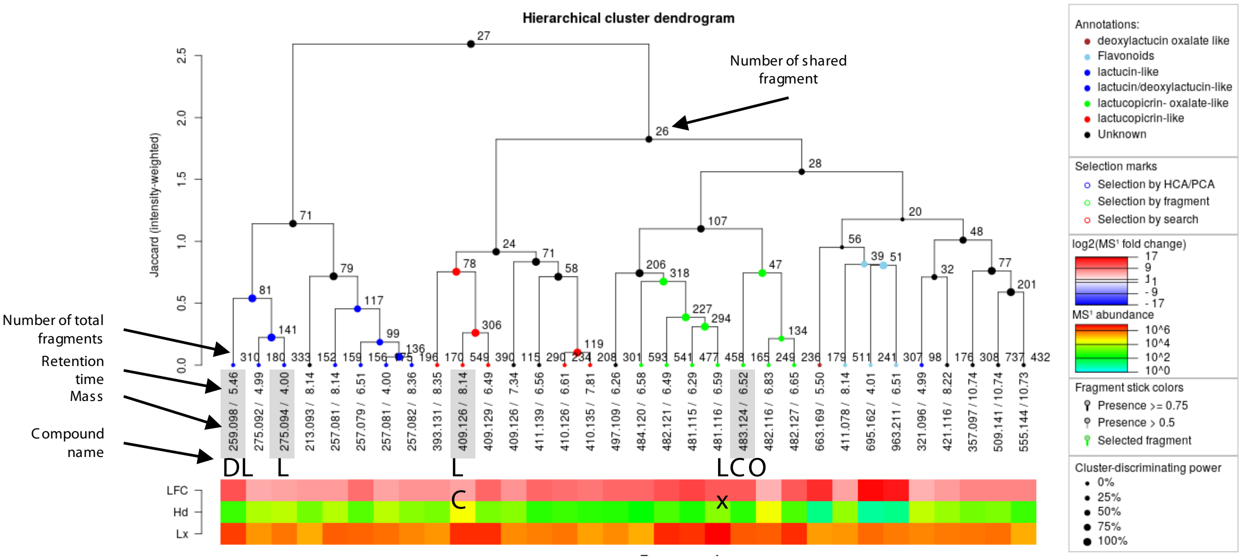

Figure S4. Excerpt of a MetFamily output focusing on sesquiterpene lactones. Hierarchical clustering of peak area from highly abundant fragment in LX and HD tissues measured by LC-MS-Pos. DL: deoxylactucin; L: lactucin; LC: lactucopicrin; LCOx: Lactucopicrin-oxalate.

A

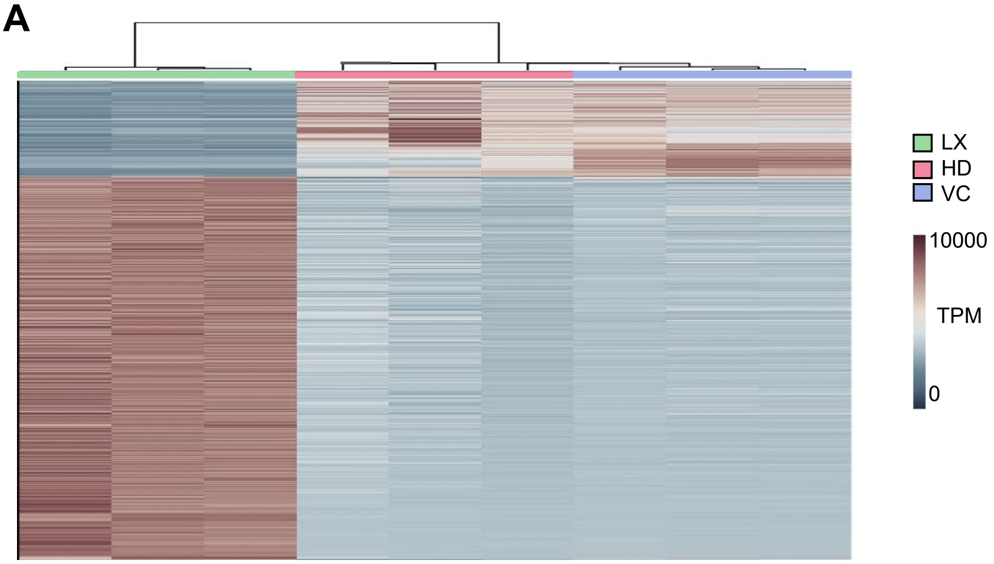

B

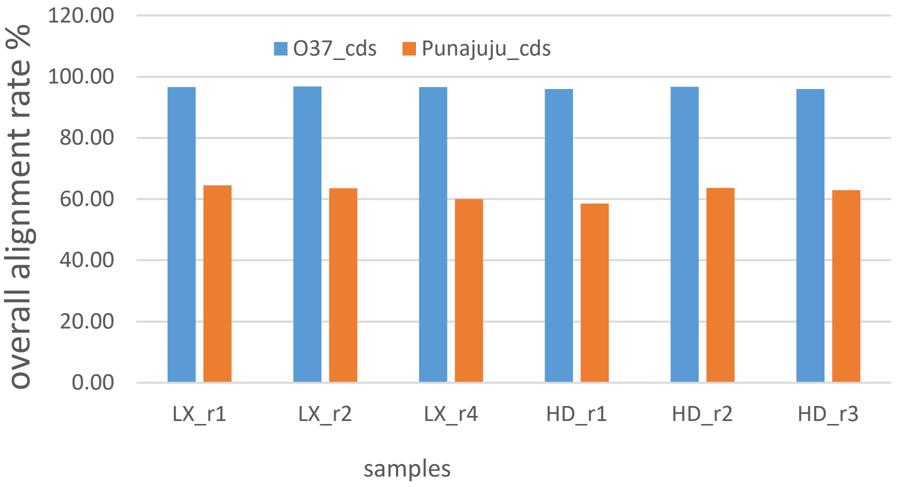

Figure S5. **Transcriptome profiling of chicory root tissues. A.** Clustering of transcriptome samples. LX: latex; HD: hypodermis; VC: vascular cylinder. **B.** Statistics of transcriptome read mapping. Graph showing the mapping of reads from our transcriptome data onto the coding sequences from the available genome (Punajuju) or to the coding sequences of Orchies37.

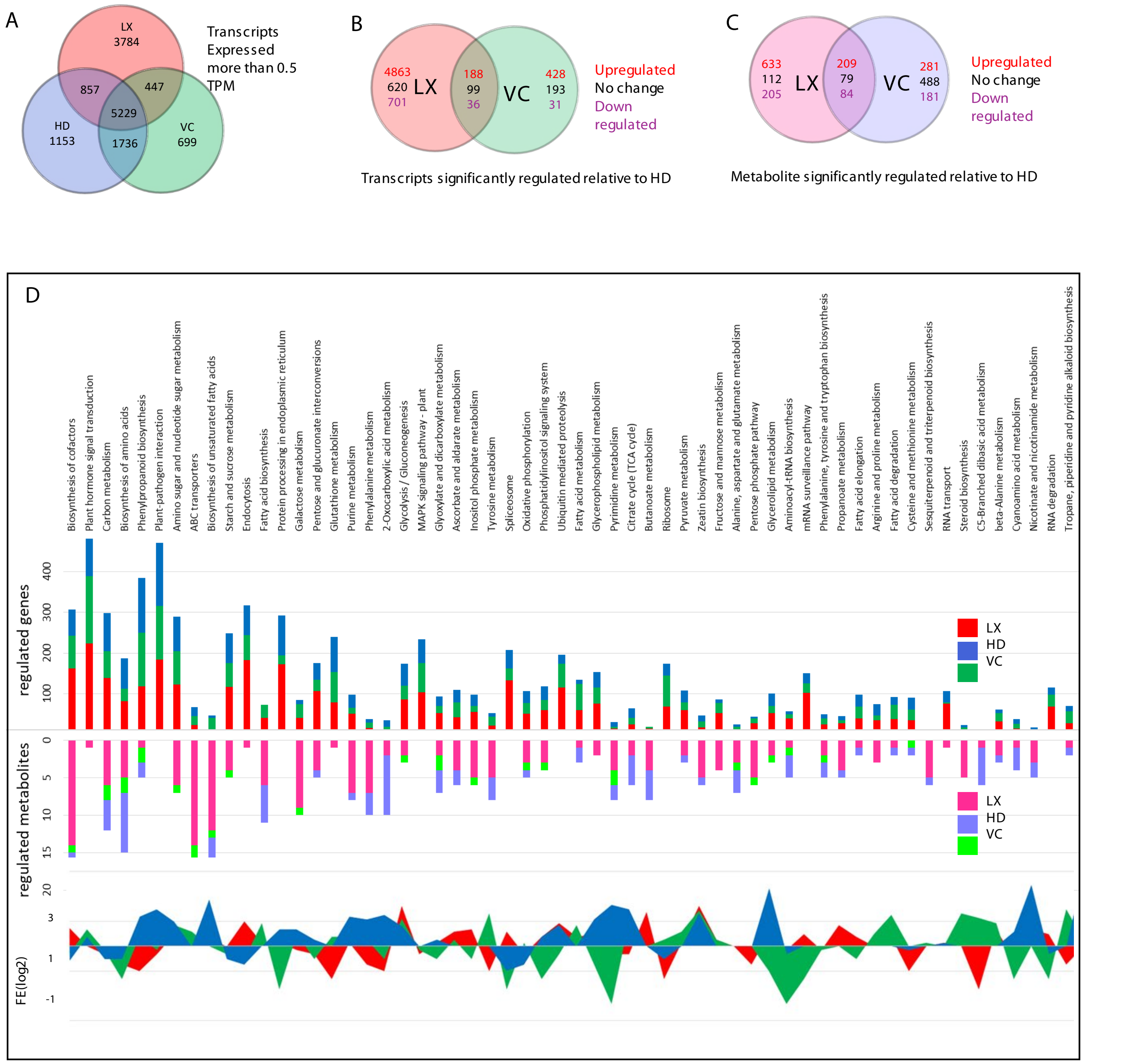

Figure S6. Overview of transcriptome data. A. Frequency pattern of regulated transcripts across different cell types. Transcripts (**B**) and metabolites (**C**) that are significantly regulated relative to HD. **D.** Tissue specific transcriptome and metabolome co-analysis of the cell types in root chicory using KEGG pathways mapping and their FE (fold enrichment) to the respected pathway.

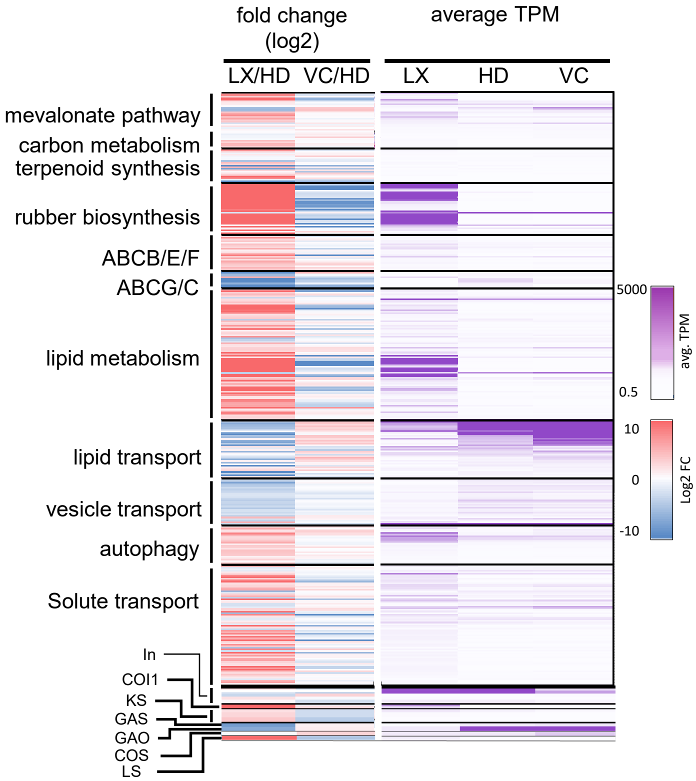

Figure S7. Expression data of selected pathways and gene families. Left panel: Log2 fold change of latex to hypodermis (LX/HD) and vascular cylinder to hypodermis (VC/HD). Right panel: gene expression values in transcript per million (TPM). ABCB/E/F: ABC transporters of families B, E and F; ABCG/C: ABC transporters of families G and C; In: Inulin biosynthesis; COI1: jasmonate receptor; KS: kauniolide synthase; GAS: germacrene synthase; GAO: germacrene A oxidase; CS: costunolide synthase; LS: lactucin synthase.

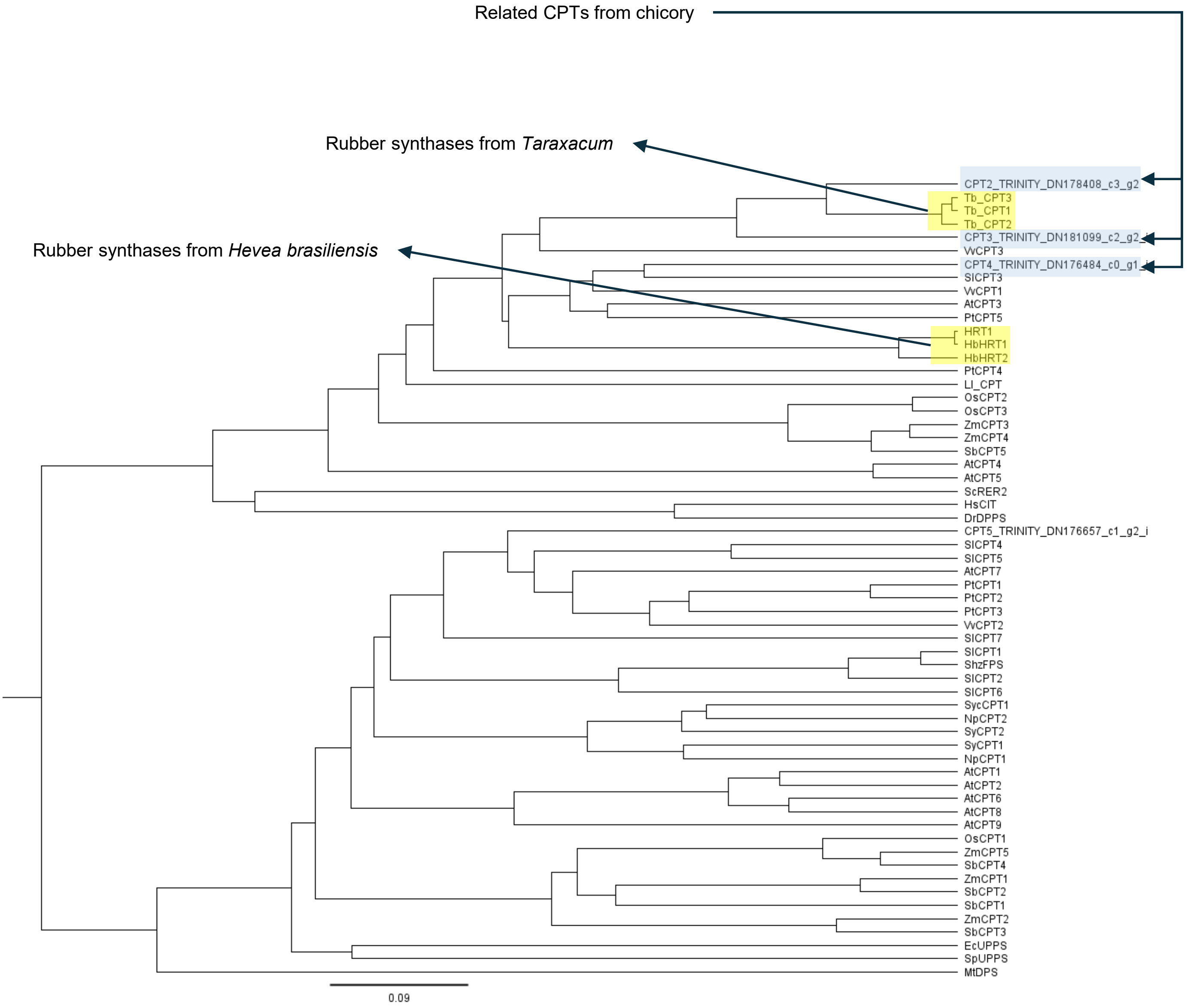

Figure S8. **Unrooted phylogenetic tree of *cis*-prenyltransferases including chicory CPT candidates and CPT genes that were described to be involved in rubber biosynthesis.** cis-isoprenyltransferase (CIT), cis-prenyltransferase (CPT), dehydrodolichyl diphosphate synthase complex (DDPS), decaprenyl diphosphate synthase (DPS), undecaprenyl pyrophosphate synthase (UPPS), z,z-farnesyl diphosphate synthase(zFPS), *Hevea brasiliensis* rubber cis-polyprenyltransferase (HRT2), *Arabidopsis thaliana* (At), *Cichorium intybus* (Ci), *Escherichia coli* (Ec), *Hevea brasiliensis* (Hb), *Homo sapiens* (Hs), *Lilium longiflorum* (Ll), *Mycobacterium tuberculosis* (Mt), *Nostoc punctiforme* (Np), *Oryza sativa* (Os), *Populus trichocarpa*(Pt), *Sorghum bicolor* (Sb), *Saccharomyces cerevisiae* (Sc), *Solanum habrochaites* (Sh), *Solanum lycopersicum* (Sl), *Streptococcus pneumoniae* (Sp), *Synechocystis* sp. (Syc), *Taraxacum brevicorniculatum* (Tb), *Vitis vinifera* (Vv) and *Zea mays* (Zm).
The tree was generated from protein sequences using Geneious v7.0 using a neighbror-joining algorithm.

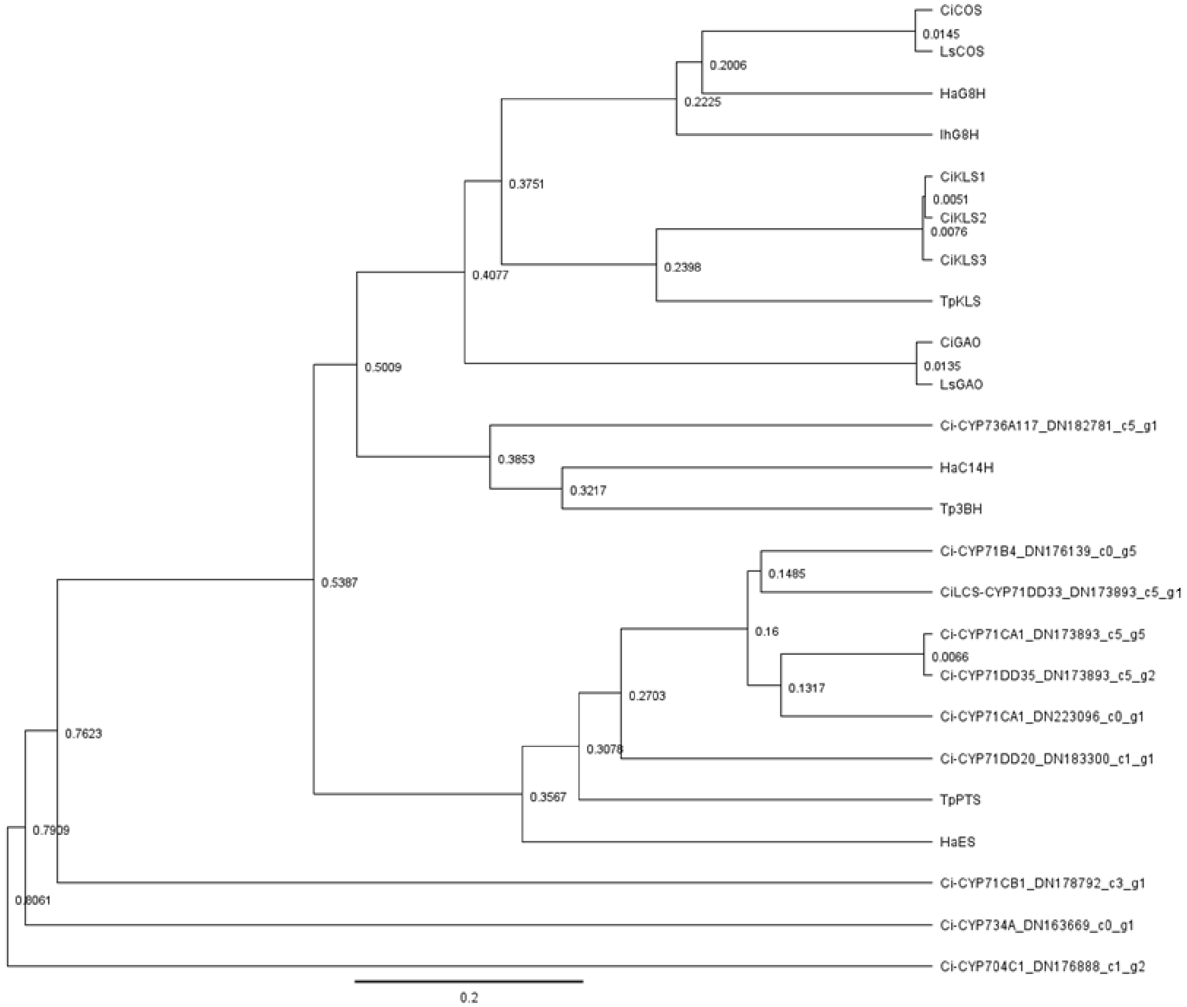

Figure S9. Unrooted phylogenetic tree of chicory cytochrome P450 oxygenases (Ci-CYP) candidates that are overexpressed in the latex. This includes other cytochrome P450 genes that were described to be involved in STL biosynthesis in the Asteraceae family including *Cichorium intybus* kauniolide synthase (CiKLS, ON456175), *Cichorium intybus* lactucin synthase (CiLCS, OP973199), *Lactuca sativa* germacrene A oxidase (LsGAO, GU198171), *Cichorium intybus* germacrene A oxidase (CiGAO, GU256644), *Lactuca sativa* costunolide synthase (LsCOS, HQ439599), *Cichorium intybus* costunolide synthase (CiCOS, JF816041), *Helianthus annuus* germacrene A acid 8-beta-hydroxylase (HaG8H, AEI59773), *Inula hupehensis* germacrene A acid 8-beta-hydroxylase (IhG8H, KR029572*), Tanacetum parthenium* kauniolide synthase (TPKLS, MF197558), *Tanacetum parthenium* parthenolide synthase (TpPTS, KC954155), *Tanacetum parthenium* 3-beta hydroxylase (Tp3BH, KC954153), *Helianthus annuus* eupatolide synthase (HaES, AEI59778) and *Helianthus annuus* costunolide 14-hydroxylase (HaC14H, MG765530). The tree was generated from protein sequences using Geneious v7.0 with the neighbour-joining algorithm.

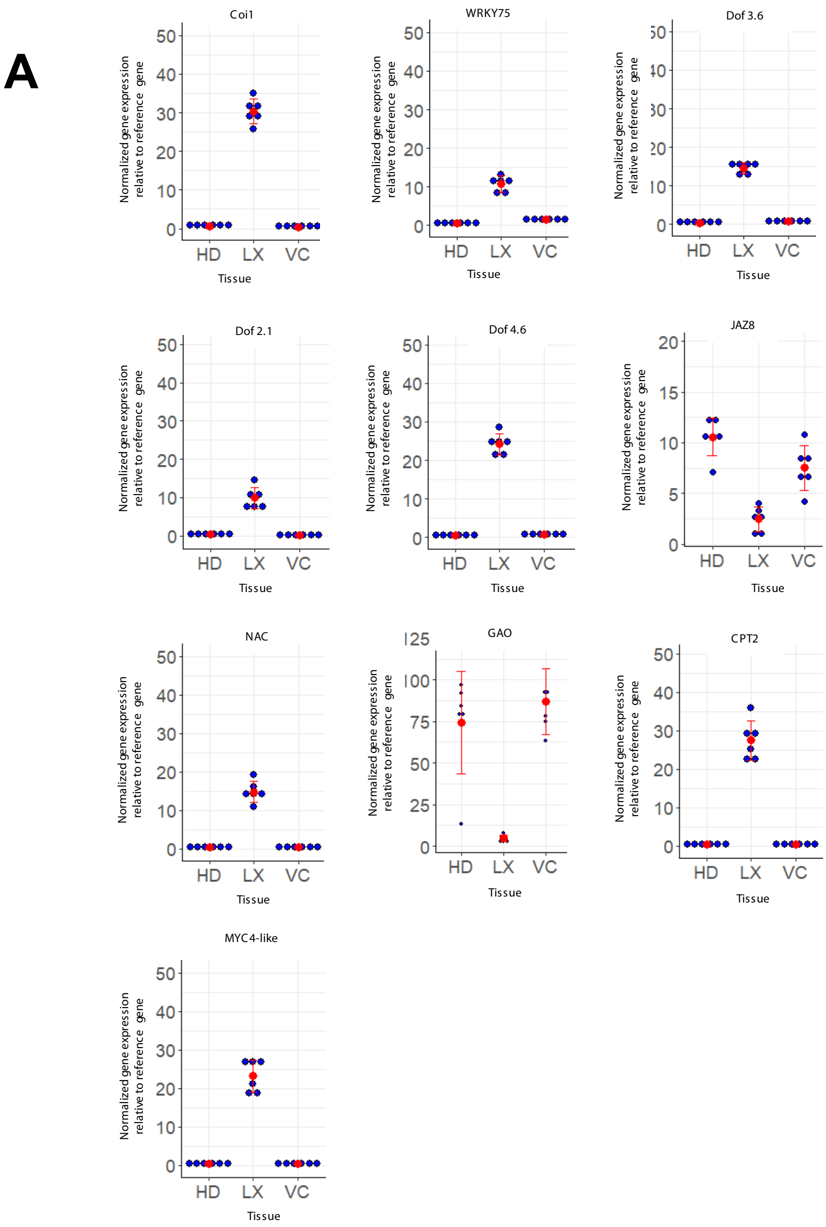
A
Coi1
WRKY75
Dof 3.6
Dof 2.1
Dof 4.6
JAZ8
NAC
GAO
CPT2
MYC4-like
Normalized gene expression relative to reference gene
HD
LX
VC
Tissue

B

Primer name	Sequence
ACTIN-Fw	CTCGAAACCGCCAAATCCAG
ACTIN-Rv	CTTTCGGCTCCGATGGTGAT
CoIP-Fw	TACCGACACCGTATTCAACTG
CoIP-Rv	TGGAGTAACACATAGCGATGG
CPT2-Fw	GTCAAGAATGTTGGTGTG
CPT2-Rv	AAGAAGCGTAATCCTCAG
DOF2.1-Fw	TCTCATCACTTCCTTCCTATCT
DOF2.1-Rv	TCACTACCGACACCAACT
DOF3.6-Fw	TACAACAACTACAGCCTTAC
DOF3.6-Rv	AGATGATGATGACGATGATG
DOF4.6-Fw	CAAGCACAGCAACAACAACTACT
DOF4.6-Rv	CTCCTCCTAACATTCCAGTCCAA
GAO-Fw	GGAAGACCGAATTGATACT
GAO-Rv	GCATATTACAACATCTATACCAT
Jaz8-Fw	CATAGAAGCAGGTGATAT
Jaz8-Rv	TTCCAGCCAATAACATTA
Myctype-Fw	TTCTTCTGCTTCTTGTTCT
Myctype-Rv	CTTCGTTGAGGTTGTCTA
NAC-Fw	TTGCCTGCTTCCATCGTT
NAC-Rv	GTATCCATCATCGCTTGCTCTA
SRPP-Fw	AGACTACTCTCAAGACTGTTGTTG
SRPP-Rv	ACCGACTCGTCCACCTTA
WRKY75-Fw	TTCTCCGAGCAGCAATAG
WRKY75-Rv	TTCTGATCTTCTTCTCACCTT

Figure S10. Verification of the tissues specific expression of a sample chicory genes by RT-qPCR.

A. Normalized expression values in three tissues (HD: hypodermis; LX: latex; VC: vascular cylinder). Actin was used as a reference gene for normalization. Values are from 3 biological replicates and 2 technical replicates. Error bars represent the standard error. **B**. List of primers used for the amplification of the cDNA fragments.

Table S1: Quantification of major sesquiterpene lactones from chicory roots

Abbreviations

L	lactucin
DL	8-deoxylactucin
LC	lactoucopicrin
LOX	lactucin 15-oxalate
DLOX	8-deoxylactucin 15-oxalate
LCOX	lactucopicrin-oxalate
LX	Latex
HD	Hypodermis
VC	Vascular cylinder

Peak areas

	HD-r1	HD-r2	HD-r3	VC-r1	VC-r2	VC-r3	Lx-r1	Lx-r2	Lx-r3
L	1285535.986	844312.7	1010865	192056.4	445657.7	105697.9	17193663.68	16816638	19079760
DL	365404.714	105698.2	226453.2	27839.8	62217.37	9137.318	5198174.261	5013480	5708471
LC	877812.2931	371280	438597	100495.1	213115.8	61563.67	9731306.624	9579329	11846585
LOX	16495522.44	9411934	18567545	4387191	9070771	2032109	30597144.16	30920472	37964971
DLOX	7544177.967	2439370	9282257	1510398	3352575	800281.4	79497134.69	53111598	61679339
LCOX	11646211.53	5465523	13790250	4106612	8799180	2148102	24893897.92	29976335	26921924

log2 fold changes

	FC_LX/HD	FC_VC/HD	Pval_LX/HI	Pval_VC/H	FClog2_LX/	FClog2_VC	FClog2_SD_LX/	FClog2_SD_VC/HD
L	17.38898	0.23670	0.001	0.009	4.09859	-2.32892	0.31	1.04
DL	28.95532	0.14220	0.001	0.112	4.68468	-3.21117	0.87	1.39
LC	21.29896	0.22230	0.004	0.101	4.30514	-2.35904	0.72	0.90
LOX	2.39494	0.34829	0.008	0.054	1.21307	-1.77746	0.44	1.08
DLOX	12.98503	0.29395	0.013	0.147	3.52472	-2.01008	0.86	1.04

Acquisition parameters

name	mz1	mz2	r.t.	DP	EP	CE	CXP
DLOX	333.1	243.1	6	35	10	35	5
LOX	349.1	241.1	4.16	35	10	35	5
LCOX	483.1	241.1	7.15	35	10	35	5
L	277.1	213.1	4.75	35	10	35	5
DL	261.1	215.1	6.63	35	10	35	5
LC	411.1	259.1	7.91	35	10	35	5

Quantification parameters

Name	Mass	Retention time
DLOX	333.1	6
LOX	349.1	4.16
LCOX	483.1	7.15
L	277.1	4.75
DL	261.1	6.63
LC	411.1	7.91

Table S6: Inulin and sugar analysis

							Total sugars after hydrolysis						Free sugars						Inulin bound sugars						
				500x	500x																			Average	
				mg/L	mg/L	mg/gDw	mg/gDw	mg/gDw		mMol/gDw	mMol/gDw	mg/gDw	mg/gDw	mg/gDw	mMol/gDw	mMol/gDw	mMol/gDw	mMol/gDw	mMol/gDw		Average		total inulin	total inulin	
			mg Dw	glucose	fructose	Glucose	fructose	Total sugars	F/G ratio	Glucose	fructose	Glucose	fructose	Sucrose	Glucose	fructose	Sucrose	Glucose	fructose	mDP	mDP	STDEV	mg/gDw	mg/gFw	STDEV
**HD-1**	**1**	**H1 50x**	**30.3**	**0.774**	**8.576**	**61.614**	**682.339**	**743.952**	**11.074**	**0.342**	**3.791**	**2.374**	**1.882**	**15.150**	**0.013**	**0.010**	**0.044**	**0.285**	**3.736**	**14.1**	**13.9**	**0.4**	**724**	**730.4**	**19**
**HD-2**	**2**	**H2 50x**	**29.7**	**0.753**	**8.303**	**61.159**	**673.975**	**735.134**	**11.020**	**0.340**	**3.744**	**1.909**	**1.492**	**15.548**	**0.011**	**0.008**	**0.045**	**0.284**	**3.691**	**14.0**			**715**		
**HD-3**	**3**	**H3 50x**	**29.9**	**0.818**	**8.749**	**65.945**	**705.424**	**771.370**	**10.697**	**0.366**	**3.919**	**2.350**	**1.594**	**14.536**	**0.013**	**0.009**	**0.043**	**0.311**	**3.868**	**13.4**			**752**		
**LX-1**	**7**	**H7 50x**	**30.3**	**0.209**	**1.099**	**16.608**	**87.466**	**104.074**	**5.266**	**0.092**	**0.486**	**1.802**	**6.342**	**17.582**	**0.010**	**0.035**	**0.051**	**0.031**	**0.399**	**13.9**	**13.8**	**1.3**	**77**	**89.5**	**15**
**LX-2**	**8**	**H8 50x**	**29.7**	**0.213**	**1.385**	**17.298**	**112.439**	**129.737**	**6.500**	**0.096**	**0.625**	**1.231**	**4.783**	**17.148**	**0.007**	**0.027**	**0.050**	**0.039**	**0.548**	**15.0**			**106**		
**LX-3**	**9**	**H9 50x**	**30.3**	**0.199**	**1.138**	**15.801**	**90.580**	**106.381**	**5.733**	**0.088**	**0.503**	**1.854**	**4.909**	**13.414**	**0.010**	**0.027**	**0.039**	**0.038**	**0.437**	**12.4**			**85**		
**VC-1**	**16**	**H16 50x**	**30.1**	**0.953**	**8.513**	**76.352**	**681.799**	**758.151**	**8.930**	**0.424**	**3.788**	**1.263**	**0.906**	**24.474**	**0.007**	**0.005**	**0.072**	**0.346**	**3.711**	**11.7**	**11.8**	**0.2**	**730**	**711.6**	**30**
**VC-2**	**17**	**H17 50x**	**30.3**	**0.888**	**7.986**	**70.633**	**635.370**	**706.003**	**8.995**	**0.392**	**3.530**	**1.094**	**1.394**	**25.608**	**0.006**	**0.008**	**0.075**	**0.311**	**3.447**	**12.1**			**677**		
**VC-3**	**18**	**H18 50x**	**30.1**	**0.957**	**8.484**	**76.682**	**679.496**	**756.178**	**8.861**	**0.426**	**3.775**	**1.132**	**1.091**	**24.499**	**0.006**	**0.006**	**0.072**	**0.348**	**3.697**	**11.6**			**728**		
